# Genomic characterization of clinical *Borrelia burgdorferi* sensu lato isolates in the Netherlands over a thirty-year period

**DOI:** 10.1186/s12864-025-12357-4

**Published:** 2025-11-27

**Authors:** Zhenghui Li, Jonathan T. Lee, Varun Raghuraman, Lorna D. Nunez, Urvi Rajyaguru, Amber Vrijlandt, Katrina E. Llamera, Lubomira Andrew, Alje P. Van Dam, Annaliesa S. Anderson, Paul A. Liberator, Li Hao, Raphael Simon, Joppe W. Hovius

**Affiliations:** 1 Vaccines, Pfizer Inc, Pearl River, NY 10965 USA; 2 Amsterdam UMC, location Academic Medical Center, Center for Infection and Molecular Medicine, Amsterdam, The Netherlands; 3 Amsterdam Institute for Immunology & Infectious Diseases, Amsterdam UMC, Amsterdam, The Netherlands; 4 National Reference Laboratory for Lyme borreliosis, Department of Medical Microbiology, Amsterdam UMC, Amsterdam, The Netherlands; 5 Department of Internal Medicine, Section of Infectious Diseases, Amsterdam UMC Multidisciplinary Lyme borreliosis Center, Amsterdam UMC, Amsterdam, The Netherlands

**Keywords:** Whole genome sequencing, Phylogenetic analysis, Borrelia afzelii, Plasmid diversity

## Abstract

**Background:**

Lyme borreliosis (LB), the most common vector-borne disease in the Northern Hemisphere, is caused by a multitude of pathogenic *Borrelia burgdorferi* sensu lato (sl) species endemic to northern, western, and central regions of Europe. We utilized whole genome sequencing (WGS) to characterize the genetic diversity of 130 clinical *B. burgdorferi* sl isolates collected from LB cases in the Netherlands between 1988 and 2023, the majority of which were *B. afzelii*.

**Results:**

WGS analysis revealed significant diversity, including 29 different multi-locus sequence types (MLSTs) across four genospecies. Plasmids from *B. garinii* and *B. bavariensis* were found to exhibit greater sequence diversity than those from *B. burgdorferi* sensu stricto and *B. afzelii*. We further characterized the *Borrelia* membrane protein antigens OspA, OspC and DbpA for sequence diversity and correlation with LB disease state.

**Conclusions:**

This large-scale genomic analysis of clinical Dutch *B. burgdorferi* sl isolates furthers our understanding of LB and may indicate potential coverage for LB vaccine candidates in clinical development.

**Supplementary Information:**

The online version contains supplementary material available at 10.1186/s12864-025-12357-4.

## Background

Lyme borreliosis (LB), the most common vector-borne disease in humans in the Northern Hemisphere, is caused by pathogenic members of the *Borrelia burgdorferi* sensu lato (sl) complex, which consists of multiple bacterial genospecies depending on the geographical region [1–3]. Within Europe, LB is endemic in the northern, western, and central regions [4]. The Netherlands, located in western Europe, has among the highest rates of recorded LB, and has observed an increase in LB cases over a 20-year period, with a more than 3-fold rise in the average number of cases per 100,000 persons per year from 1994 to 2014 [5]. A large-scale, population-based cohort study conducted between 2015 and 2019 found that Dutch LB incidence rates remained elevated, ranging from 111 to 131 per 100,000 persons per year [6]. During this time, over 1 in 25 (4.4%) inhabitants of the Netherlands general population were found to have *B. burgdorferi* sl-specific antibodies [7]. In addition, a sequencing study of Dutch *Borrelia*-positive ticks found a high density of rare genetic variants, suggesting genetic population expansion for *Borrelia afzelii* and *Borrelia garinii* [8], the predominant LB-causing species in the Netherlands [9]. These results are consistent with the reported increase in disease incidence in this region of Europe.

Over the last decade, empowered by the rapid advancement of next-generation sequencing (NGS) and sequence analysis algorithms, whole genome sequencing (WGS) has enabled a better understanding of *Borrelia* diversity, with more studies utilizing WGS data to understand *Borrelia* genome variation and population structure [10–12]. However, since microbiological recovery of *Borrelia* bacteria from patients with LB is technically challenging, there is far more known with respect to *Borrelia* populations in *Ixodes* ticks compared to those associated with human disease. Epidemiological surveillance of *Borrelia* in the Netherlands has relied largely on studies of sampled *Ixodes ricinus* ticks (nymphal and adult) and rodents [9, 13–16], which can include analysis of non-human pathogenic genospecies such as *B. valaisiana* and *B. turdi* [9]. To date, reports that have analyzed the genetic diversity of Dutch *Borrelia* directly from infected human tissue have been limited to only few isolates and traditional typing [17] or multilocus sequence type (MLST) analysis [18]. NGS has emerged as a promising alternative to standard PCR-based typing that can characterize *Borrelia* in high-throughput. WGS enables in-depth analysis of both the chromosome and *Borrelia* plasmids, which carry critical antigen-encoding genes relevant to LB diagnostics and vaccine development.

For this purpose, we compiled a database of assembled *Borrelia* genome sequences from 130 clinical isolates over a 30-year period in the Netherlands. We evaluated genetic diversity and relatedness of these isolates, comparable to isolates collected elsewhere in the world, by performing core genome alignment and genome-wide phylogenetic analysis. Based on the WGS analysis of four pathogenic *Borrelia* genospecies (*B. burgdorferi* sensu stricto (ss), *B. garinii*, *B. afzelii*, and *B. bavariensis*) found in western Europe, we examined sequence differences in multi-locus sequence type (MLST) genes as well as those in three widely studied surface antigens: outer surface protein A (OspA), OspC, and decorin binding protein A (DbpA). These virulence factors, expressed in either tick (OspA) [19] or mammalian stages (OspC, DbpA) [20–23], are protective antigens as they have been shown to be effective vaccines in animal models of LB [24–27]. These proteins are of further interest, having also served as markers in the serological diagnosis [28, 29] or typing of *B. burgdorferi* sl [30–32]. In addition, we analyzed seven genes of interest, implicated in *Borrelia* immune evasion, for their sequence conservation in this collection.

## Methods

### Sources of *Borrelia* isolate collections

*Borrelia* genomes used for this study were sourced from a collection of isolates of *B. burgdorferi* sl from Dutch LB cases with distinct clinical manifestations, collected at Amsterdam UMC, location AMC, over a 30-year period from 1988 to 2023 (*n* = 130) (Table S1, PRJNA1041728). Information on a subset of the patients has been published previously [33, 34]. Metadata for all isolates (e.g., disease stage, year of isolation, genospecies, etc.) can be found in Table S1. For comparison, this dataset was evaluated against publicly available genome sequences (*n* = 170) from the PubMLST *Borrelia* isolates database (https://pubmlst.org) and GenBank (Table S2) sourced from humans, animals, and ticks across multiple continents (North America, Europe, and Asia). Accession numbers of the externally sourced isolates are included in Table S2.

### Bacterial growth and whole genome sequencing

MKP (Modified Kelly-Pettenkofer) media prepared in-house was used for cultivation of *B. afzelii*,* B. garinii*,* B. bavariensis* and *B. spielmanii* isolates frozen stock vials, whereas BSK-H (modified Barbour-Stonenner Kelly, Sigma-Aldrich) media was used for the culture of *B. burgdorferi* ss. Cultures were incubated at 34° C for 5 days and monitored for bacterial growth by darkfield microscopy. Bacterial cell lysis and DNA extraction was performed using the Genfind V3 DNA isolation protocol (Beckman Coulter Life Sciences cat# C34881), as previously described [35]. The extracted genomic DNA was then sequenced on the Illumina MiSeq, with 2 × 300 bp paired-end sequencing chemistry following a slightly modified protocol, as previously described by Jones et al. [36].

### Genotype characterization, phylogenetic analysis, and pairwise SNP analysis

For NGS reads from each isolate, *de novo* genome assembly was performed using the CLC Genomic Workbench (v.21) with default settings. The BIGSdb (Bacterial Isolate Genome Sequence Database) [37] was implemented to extract and characterize genetic information corresponding to targets of interest using BLAST: 8 housekeeping genes for MLST (multi-locus sequence type), and three surface proteins (*OspA*, *OspC*, *DbpA*). Unique alleles and protein variants were assigned numerical identifiers using BIGSdb (Table S1, S2). Gene-specific phylogenetic trees were generated using MEGA11 [38]. Whole-genome core alignments and phylogenetic analysis were performed using Parsnp [39], a rapid genome aligner from Harvest suite, with customized phylogenetic tree visualization feature.

To calculate pairwise SNP (single nucleotide polymorphism) distances between two *B. burgdorferi* sl strains, raw whole genomes sequence data were aligned to reference genomes for *B. garinii* (GCA_003814405.1) and *B. bavariensis* (GCA_003814425.1) using the CLC Genomics Workbench. Variant calling was performed with > 90% frequency for SNP calling. SNP distances were calculated using the *create SNP tree* function in CLC Genomics Workbench Microbial Genomics Module with default parameters.

### Plasmid comparative genomic analysis

To estimate plasmid coverage, a BLAST local alignment (v2.13.0) was performed for each assembled genome against the plasmid reference sequence. The reference plasmid sequences of four *Borrelia* genospecies were obtained from annotated reference genomes for each genospecies studied: *B. burgdorferi* ss: B31_NRZ (GCA_002151505.1); *B. afzelii*: K78 (GCA_000962775.1); *B. garinii*: 20,047 (GCA_003814405.1); and *B. bavariensis*: PBi (GCA_003814425.1). To illustrate the plasmid sequence conservation across isolates, BLAST alignment results were separated into 1 kb bins and plotted as disc diagrams using the R circlize package (v0.4.15), in which each ring represents similarity of an assembled genome compared to the reference plasmids.

### Sequence conservation analysis of genes involved in immune evasion

Gene sequences from each isolate were retrieved using BLASTN with *B. burgdorferi* ss B31 as the reference genome and translated to peptide sequences. BLAST hits with incomplete reading frames were ignored. Pairwise amino acid sequence identity within each gene was calculated within each serotype as well as against B31. This calculation was not performed when only a single unique protein variant was identified within a serotype.

## Results

### Clinical *Borrelia* strain collection and WGS data

In this study, a total of 130 Dutch *Borrelia* isolates collected from LB patients were cultured, sequenced, and analyzed. On average, 2.5 million paired-end reads (2 × 300 bp) were generated per sample (Table S1), and the number of *de novo* assembled contigs varied between 62 and 1270. The median assembled genome size was approximately 1.4 Mb, in which the chromosome was generally present as single contig with a length of approximately 900Kb and the remaining content being plasmid sequences. The median chromosome coverage depth ranged between 13× and 475× (Table S1). Using assembled genome contigs, each of the strains was genotyped using the standard *Borrelia* MLST scheme [40]. We identified a total of 29 MLSTs in this dataset, with thirty-six isolates (27.7%) that had a novel MLST (Table S1).

**Fig. 1:**
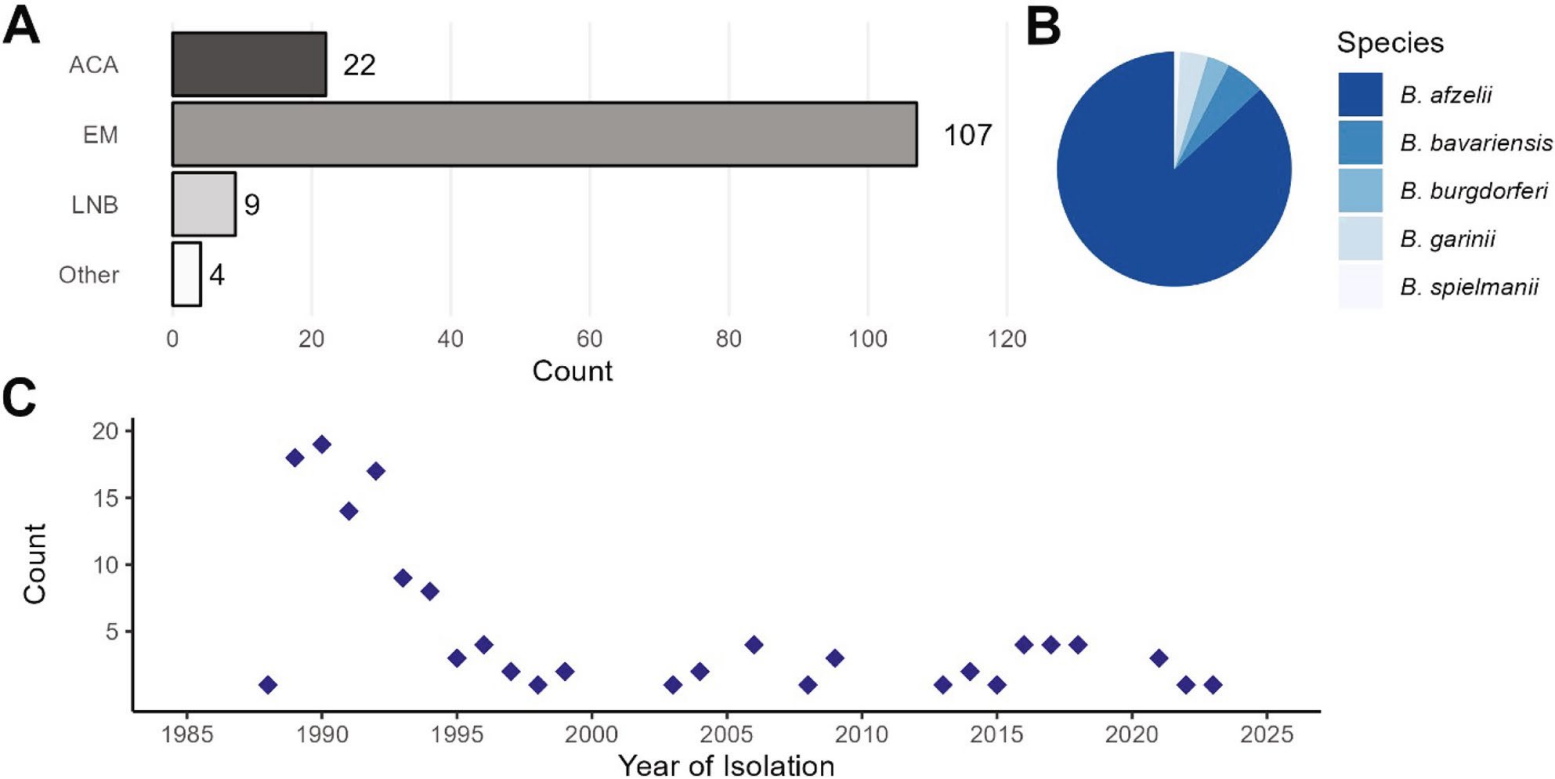
Host site and genospecies distribution of 130 Dutch *Borrelia* strains. **A** Bar chart depicting the number of different LB clinical manifestations of Dutch patients from which *Borrelia* isolates of this collection were sampled. Isolates (*n* = 130) were largely collected from patients exhibiting EM (*n* = 107, 75.9%), with other LB manifestations at lower frequency. Some patients exhibited multiple disease states, and two patients experienced multiple EMs. **B** The sequenced Dutch isolates spanned five genospecies, with *B. afzelii* being the most prevalent genospecies collected (*n* = 113, 86.9%). **C** Plot depicting the number of isolates collected per year at Amsterdam UMC in Amsterdam, Netherlands, over a 35-year period from 1988 to 2023. EM, erythema migrans; ACA, acrodermatitis chronica atrophicans; LNB, Lyme neuroborreliosis

Aside from three isolates which were collected from cerebrospinal fluid (CSF), all isolates were recovered from skin biopsies of Dutch patients with erythema migrans (EM) or acrodermatitis chronica atrophicans (ACA) (Table S1). A total of 107 patients (82.3%) had at least one EM, 22 patients (16.9%) had an ACA, 9 patients (6.9%) had Lyme neuroborreliosis (LNB), and 4 patients (3.1%) exhibited other LB manifestations (Lyme arthritis, carditis or multiple EM) (Fig. 1A). Of these, 12 patients (9.2%) had multiple LB manifestations, with EM + LNB being the most prevalent combination (*n* = 7). The majority (86.9%) of isolates in the Dutch collection were *B. afzelii* (Fig. 1B). Other genospecies included *B. bavariensis* (*n* = 7, 5.4%), *B. garinii* (*n* = 5, 3.8%)) and *B. burgdorferi* ss (*n* = 4, 3.1%), as well as a single *B. spielmanii* isolate (0.8%). Clinical samples were collected across four decades (1988–2023) (Fig. 1C). Approximately two thirds (68.5%) of the Dutch isolates were collected in 1995 or earlier, and a collection of more contemporary isolates was acquired between 2013 and 2023 (16.2%).

### Genome-wide phylogenomic analysis of *Borrelia* isolates

To understand the relationship of the Dutch clinical *Borrelia* isolates to those previously published, we compared them to 170 *Borrelia* genome sequences from public databases (PubMLST and GenBank; Table S2). This publicly available isolates were collected from 19 countries across North America, Europe, and Asia, between 1981 and 2017. A total of 11 genospecies were represented (49.4% *B. burgdorferi* ss, 21.8% *B. garinii*, 19.4% *B. bavariensis*, and 4.1% *B. afzelii*) (Table S2).

**Fig. 2:**
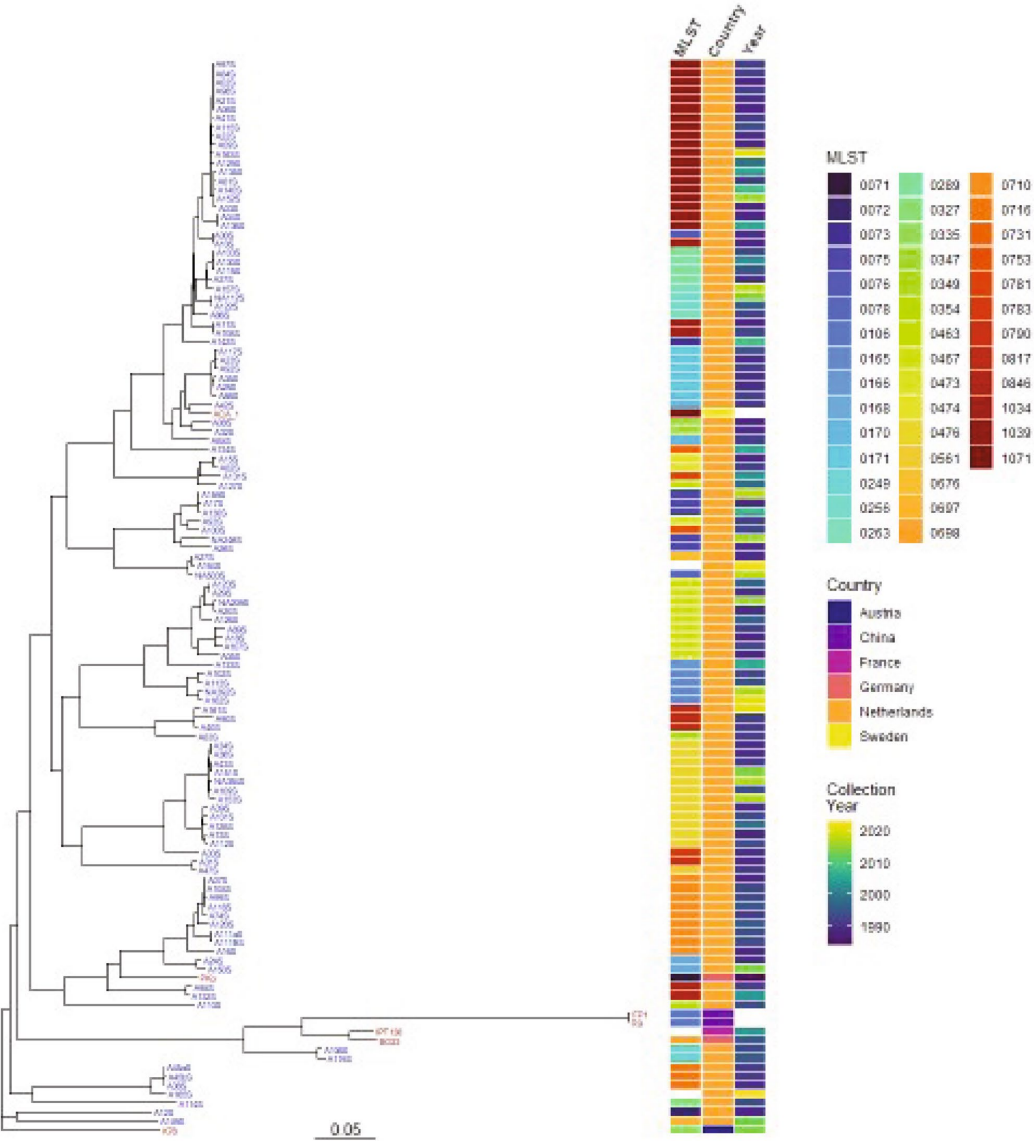
Genome-based phylogenetic trees of *B. afzelii*. Phylogeny and metadata are shown for both the Dutch *Borrelia* strain collection (blue, *n* = 113) and publicly available genomes (red, *n* = 7) of *B. afzelii*. Isolate MLST, country of collection, and collection year are color coded according to their respective color keys. Blank spaces indicate missing metadata or an incomplete MLST.

The Dutch *B. afzelii* isolates spanned multiple small, closely related clusters correlating with their MLST, but not their year of collection. For instance, MLSTs 1039, 289, 171, 75, 476, 710, 467, and 165 all formed individual clusters (Fig. 2). Isolates from other countries in the public database were interspersed with those collected from Dutch LB cases. One group of five isolates, including the *B. afzelii* BO23 strain, was found to be more diverged from the remainder of the Dutch collection.

In contrast, the assembled genomes of *B. burgdorferi* ss isolates (*n* >120) were primarily grouped into four large groups with longer branch lengths (Figure S1). Within these groups, isolates formed closely related clusters based on MLST. For instance, isolates with MLSTs 1, 29, 3, and 32 each formed distinct subgroups with short branch lengths (top of the Figure S1). Although the Dutch *B. burgdorferi* ss isolates did not cluster with one another, one isolate was found to be most closely related to *B. burgdorferi* ss collected from Germany and Italy. The phylogenetic tree generated from assembled genomes of *B. garinii* isolates (*n* >50) exhibited a different pattern with even longer branch lengths (Figure S2). Unlike *B. afzelii* and *B. burgdorferi* ss, *B. garinii* spans multiple OspA serotypes [32] and in silico types (ISTs) [35]. Accordingly, *B. garinii* collected from the Netherlands clustered with *B. garinii* isolates from other locations that shared the same OspA IST. Finally, two clusters were formed in *B. bavariensis*: one with longer branches consisting solely of public IST9 and IST10 isolates, and the other a tight cluster with short branch lengths represented entirely by OspA IST4 isolates, including all the Dutch *B. bavariensis* isolates (Figure S3). The observation of two major clusters in *B. bavariensis* phylogeny is consistent with a prior study [10].

In several instances, multiple isolates were collected from the same subject at different body sites. This was the case for subjects A77 and A91 (Table S1). We performed pairwise SNP distance calculation to investigate sequence similarity of isolates from the same patients. For the three *B. garinii* isolates collected from subject A77, the SNP distances among the three biopsies was *≤* 11. In contrast, the SNP distance between A77 isolates and biopsies collected from other subjects was *≥* 5,719. *B. bavariensis* isolates collected from skin and CSF of subject A91 were genetically identical, having a SNP distance of zero, compared to a distance of *≥* 192 relative to isolates from other subjects. When compared to the SNP distances between subjects, these results indicated that the multiple biopsies yielded the same strain in each subject.

### Plasmid diversity within *B. burgdorferi* sl

**Fig. 3:**
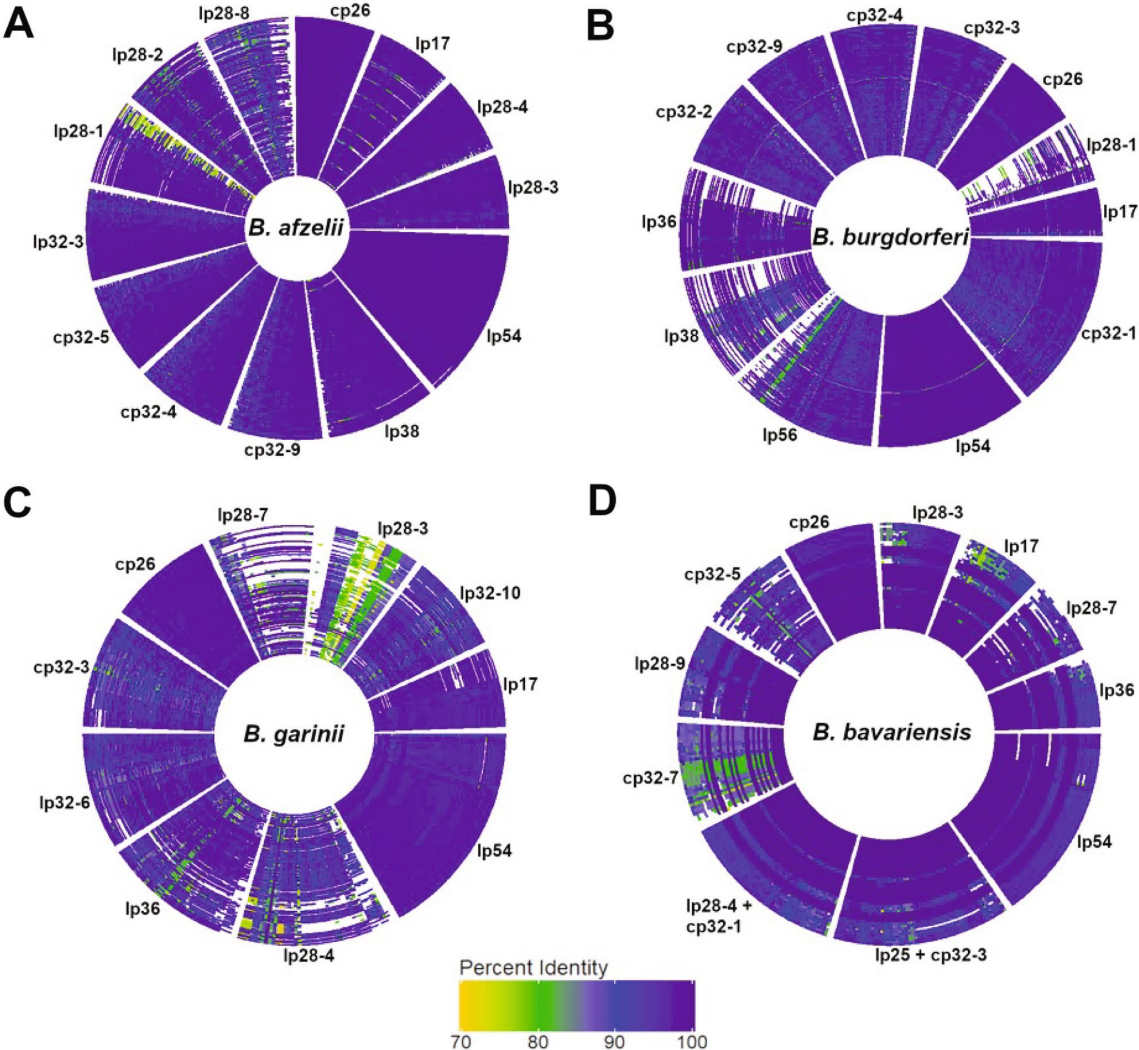
Plasmid coverage within *Borrelia* genospecies. BLAST coverage breadth across known plasmids for isolates belonging to each of four genospecies: (**A**) *B. afzelii*, (**B**) *B. burgdorferi* ss, (**C**) *B. garinii*, and (**D**) *B. bavariensis* from both the Dutch *Borrelia* strain collection and public *Borrelia* isolates. 70–100% identity to the species reference plasmid sequence is indicated on a color scale (yellow to blue, blue indicating 100% sequence identity). Regions depicted in white indicate 0% BLAST alignment. Reference plasmid sequences for each genospecies are provided in the Materials and Methods. Lp, linear plasmid; cp., circular plasmid

Given that roughly one-third of the *Borrelia* genome is encoded on circular (cp.) and linear (lp) plasmids, we next sought to determine the plasmid sequence diversity across the Dutch and the public isolates. Sequence identity to the reference sequences for each isolate are depicted in Fig. 3. Overall, the plasmids from *B. afzelii* were more consistently similar to their respective species reference sequence, compared to plasmids from *B. burgdorferi* ss, *B. bavariensis* and *B. garinii*. Additionally, the intra-genospecies variation for the plasmid coverage of *B. afzelii* was also much lower than that of the other genospecies, which is consistent with patterns observed from our genome-wide phylogenetic analysis.

Consistent with previous observations [41, 42], lp54 and cp26 were highly conserved at >95% identity across almost the entire plasmid sequences in all four genospecies examined. The exception was non-IST4 *B. bavariensis* (Fig. 3D, outer ring), which exhibited more sequence divergence on lp54. Still, this group had less sequence divergence in lp54 compared to other plasmids. In particular, the cp32 series plasmids were highly diverged in non-IST4 *B. bavariensis*, with sequence variation spanning the full length of the plasmid.

The lp28 series plasmids were found to be some of the most variable, from which large sections spanning multiple kilobases (kb) were found to exhibit high sequence divergence. This was particularly notable in *B. garinii* and *B. burgdorferi* ss. Many *B. garinii* isolates exhibited low or no identity to the reference sequence across nearly the entirety of lp28-3, lp28-4, and lp28-7. Similarly, large portions of lp28-1, lp36, and lp38 had no match to the reference sequence across *B. burgdorferi* ss isolates.

### Sequence diversity of OspA and DbpA on lp54

OspA is an outer surface lipoprotein crucial for the colonization of the *B. burgdorferi* sl in the tick [19]. Among Osp proteins A through D, OspA has been shown to exhibit the lowest sequence variability compared to other *Borrelia* outer surface proteins [43]. Eleven unique OspA protein variants were observed in the Dutch strain collection, with each variant found to be OspA IST-specific. OspA was highly conserved in *B. afzelii*: only four OspA variants were observed across the 112 *B. afzelii* isolates, 82% (*n* = 92) of which were variant 7. This was also the only full-length OspA variant found in *B. afzelii* isolates from the wider genome collection (Table S2). Among the other genospecies in the Dutch isolates, there were two OspA variants among *B. burgdorferi* ss isolates belonging to OspA IST1, three variants among *B. garinii* under OspA IST5-6, and only a single OspA variant across *B. bavariensis* isolates equating to OspA IST4. In contrast, more OspA diversity was observed among the 170 genomes from public data sources as 49 unique OspA variants were identified. This sequence variation was largely observed in *B. garinii*, for which 21 (43%) OspA variants were detected across seven OspA ISTs (IST3, IST5-8, IST11-12). These results are consistent with a previous analysis [35], where we determined that high OspA diversity and multiple distinct OspA ISTs were found among *B. garinii*, but less so for *B. afzelii* and *B. burgdorferi* ss.

**Fig. 4:**
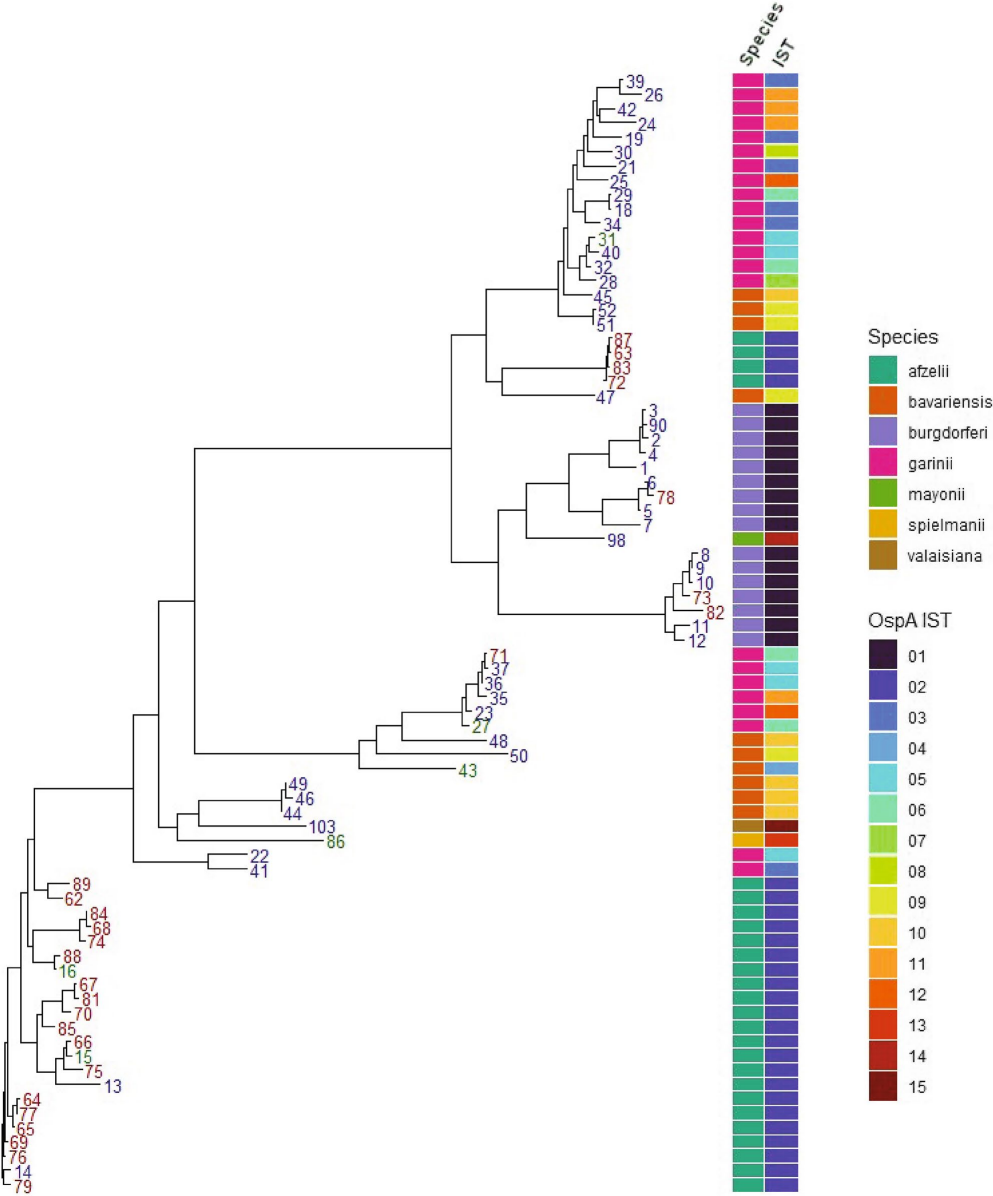
Phylogenetic Tree of DbpA variants. Protein-based phylogeny of DbpA in both the Dutch *Borrelia* strain collection (blue, *n* = 115) and public *Borrelia* isolates (red, *n* = 142). *Borrelia* genospecies and OspA IST are color coded according to their respective color keys. Green tip labels indicate variants found in both Dutch and public isolates. IST, *in silico* type

Unlike OspA, decorin-binding protein A (DbpA) is expressed during mammalian infection and is a dispensable virulence factor associated with enhanced binding of the extracellular matrix [23]. A total of 32 unique DbpA protein variants were identified among the Dutch isolates, with 6 of these also present in the publicly sourced genomes. These were compared to 46 additional DpbA variants found among the public isolates. Phylogenetic analysis (Fig. 4) revealed that, although sequences appeared to cluster by species, DbpA variants from the Netherlands did not necessarily form their own unique groups. DbpA variants from *B. afzelii* occurred in multiple divergent clusters, with variants 14 and 79 furthest removed from the other sequences. Twenty related DbpA variants found in *B. afzelii*, mostly associated with the Dutch isolates, formed a large phylogenetic group towards the bottom of the tree. Notably, four other DbpA variants (72, 83, 63, and 87) formed a phylogenetic group located in the middle of the tree. In contrast, variants associated with *B. burgdorferi* ss formed a monophyletic group on the tree. DbpA variants associated with other *Borrelia* species including *B. bavariensis*, *B. garinii*, *B. mayonii*, and *B. spielmanii* were all interspaced on the phylogenetic tree. In *B. garinii* and *B. bavariensis*, there was not a correlation between OspA IST and DbpA despite both genes occurring on the same plasmid lp54.

### OspC diversity of the *Borrelia* strains from the Netherlands

OspC is an additional *Borrelia* virulence factor that is required to establish infection in mammals [20, 22]. Compared with OspA, OspC is much more genetically and phenotypically heterogeneous, especially for *B. burgdorferi* ss and *B. afzelii* strains [31]. A total of 25 unique OspC variants were observed in the Dutch strain collection, with the most common sequence (variant 17) found in 32% of the isolates (41 of 130 isolates). When compared to the *B. burgdorferi* ss isolate B31 OspC sequence, the pairwise amino acid sequence identities of these OspC variants ranged from 64% (variant 55) to 79% (variant 72). In contrast, more diversity was observed in the *Borrelia* isolates curated from public databases where a total of 71 OspC variants were observed with the most prevalent OspC variant found in 11% of isolates (19 of 170 isolates). The pairwise amino acid sequence identity to B31 OspC was also more varied, ranging from 50% to 100%. A total of seven variants (16, 17, 19, 32, 36, 53, and 71) were shared among both genome collections.

**Fig. 5:**
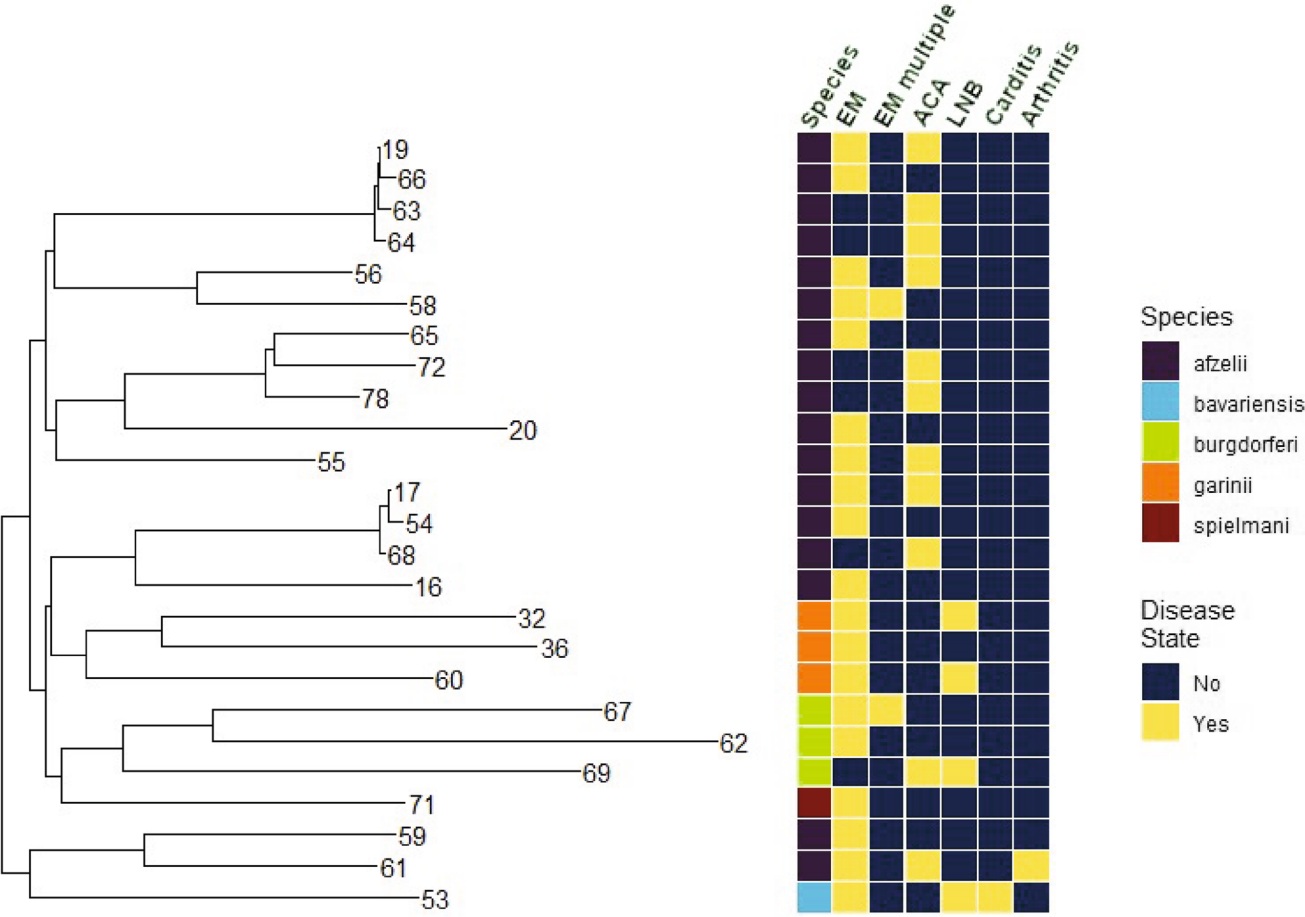
Phylogenetic tree of OspC variants. Protein-based phylogeny of OspC sequences from the Dutch *Borrelia* strain collection (*n* = 128). Disease state is depicted in yellow if the OspC variant was found in at least one isolate from a patient that exhibited that LB clinical manifestation. *Borrelia* genospecies is also color coded according to the color key. EM, erythema migrans; ACA, acrodermatitis chronica atrophicans; LNB, Lyme neuroborreliosis

Next, we combined OspC variant sequences from the Dutch and public *Borrelia* isolates as well as the publicly available OspC sequences reported by Di et al. [44], to construct a OspC variant phylogenetic tree for the Dutch isolate collection (Fig. 5). The OspC sequences were found to group by genospecies, with the *B. burgdorferi* ss OspC variants 62 and 67 clustering with the *B. burgdorferi* ss sequences previously described by Di et al. [44]. Among isolates with associated patient data, multiple OspC variants were found in more than one disease state: variants 17, 19, 55, 56 and 61 were associated with *Borrelia* isolates identified in both EM and ACA patients.

### Sequence conservation of the genes involved in immune evasion

We investigated the sequence conservation of seven genes reported to be involved in the evasion of the host immune response by *Borrelia* spp. (Table S3). As expected, pairwise amino acid sequence identities for OspA IST1 isolates to the *B. burgdorferi* ss B31 reference genome was high, ranging from 97.1% to 100% across these seven genes. The OspA IST2 *B. afzelii* isolates showed clear divergence from the B31 reference, with pairwise amino acid sequence identities ranging from 71% to 99.5%. The remaining OspA IST4, IST5 and IST6 isolates also showed considerable differences compared to the B31 reference (Table S3).

We next sought to investigate sequence diversity within each OspA IST. Amino acid sequences for all seven genes were found to be highly conserved within each OspA IST, but divergent from the B31 reference. Pairwise identity ranged from 98.6% to 99.9% in OspA IST2. However, only single unique protein variants were observed in OspA IST4 and IST5 for each of the seven genes.

## Discussion

Surveillance of isolates from human cases has the potential to be an informative tool for LB vaccine development in ensuring broad coverage against disease-causing strains. Previous genome characterizations of *B. burgdorferi* sl in Europe have focused on select genospecies and smaller sample sizes, comprising 4 [45], 8 [41], 14 [42], and 30 isolates [10]. In this study, we have leveraged WGS of 130 clinical isolates, collected from Dutch LB cases, in conjunction with >170 publicly available genomes (>300 total) to characterize sequence diversity in six pathogenic *B. burgdorferi* sl species across hundreds of isolates from a broad geographic range (North America and Europe). In doing so, we determined the genetic relatedness of Dutch *Borrelia* species, predominantly *B. afzelii* [9], to those collected elsewhere in the world. Furthermore, we associated protein variants of three known virulence factors (OspA, OspC, and DbpA) to multiple LB disease states.

Based on the sequence analysis of the intergenic spacer region of the 5 S and 23 S ribosomal RNA gene, clinical *B. afzelii* and *B. burgdorferi* ss collected from the Netherlands diverged significantly from the *Borrelia* collected from other European countries. Previous work by Coipan et al. [18] reported that the most commonly observed MLST among Dutch *B. afzelii* was 1071. In contrast, the most prevalent MLST observed in our collection of Dutch *B. afzelii* was 1039 (*n* = 19, 14.6%). Interestingly, based on the MLST results, MLST 1071 described in Coipan et al. is identical to sequence type 1039 annotated in PubMLST and reported accordingly in this manuscript. This difference was caused by Coipan et al. having independently assigned a MLST in their manuscript if it had not been previously reported at the time of publication.

Subsequent work by Gallais et al. [46], which compared the diversity of MLSTs across multiple European countries, showed that MLSTs 1039 (previously referred to as 1071), 476 and 171 are most frequently observed in clinical *Borrelia* isolates from the Netherlands whereas MLST 347 is most frequently found in France and MLST 71 in Germany. Our analysis largely corroborates with Gallais et al., as MLSTs 1039 (*n* = 19), 476 (*n* = 12), 467 (*n* = 6) and 171 (*n* = 6) were the most prevalent types found in the Dutch *B. afzelii* isolates. In addition, Gallais et al. utilized pairwise fixation indices to demonstrate that Dutch *B. afzelii* isolates differed moderately when compared to German, Slovenian, and Austrian isolates [46]. Although we did not sequence *B. afzelii* isolates from Germany, Slovenia or Austria in this study, our phylogenomic tree suggests that the Dutch *B. afzelii* isolates are distinct from *B. afzelii* found elsewhere in Europe. For instance, core genomes of isolates with the primary MLSTs found in the Dutch *B. afzelii*, 1039 and 476, are far removed from those with MLST 347, a type commonly found in France [46].

Plasmids tend to be structurally variable and can be difficult to reassemble or analyze from sequenced data without specialized techniques that factor read coverage, overlap, and/or expected gene content. To reduce error, many assembly tools recommend long-read data for analysis of shorter plasmids as best practice. In contrast, the *Borrelia* genospecies within this study possessed lengthy plasmids comprising around a third of the length of the total genome [47]. Thus, while areas of high conservation within isolates can be confirmed, more variable regions were difficult to characterize. Furthermore, the natural variation within a given genospecies can potentially make it harder to determine whether subgroups of similar plasmid conservation exist among isolates. For instance, OspA IST4 *B. bavariensis* isolates were largely consistent in their plasmid BLAST coverage, but a subgroup of isolates possessed greater variation in lp28-9 and lp32-7. This could potentially be unnoticed in a smaller dataset. Novel plasmid sequences are also not targeted by the plasmid analysis workflow. The use of short-read data for long plasmid sequences limits the use of tools to precisely examine plasmid sequences without assembly and necessitates the use of plasmid references to compare properties.

Located on the same lp54 plasmid as OspA, DbpA is a surface-exposed adhesin that facilitates bacterial attachment to host proteoglycan decorin [23] and promotes the colonization of the bacteria in a mammalian host. DbpA has been investigated as a potential Lyme vaccine antigen; however, DbpA is highly variable compared to OspA, and furthermore, antibodies raised by DbpA, immunization provided insufficient protection from *Borrelia* infection in a mammalian model [48]. DbpA has also been evaluated as a target for LB serodiagnosis; however, its high frequency of polymorphism further limits its diagnostic value [28, 49]. Our findings largely recapitulate previous work which has shown the diversity of the DbpA among *B. burgdorferi* sl.

Although multiple members of the *B. burgdorferi* sl family are the causative agents of LB, clinical manifestation tend to vary by genospecies [50, 51]. For example, *B. afzelii* is associated with the skin manifestation ACA [52] while *B. garinii* and *B. bavariensis* have been linked to LNB [18] and may cause peripheral and central nervous system abnormalities. In contrast, *B. burgdorferi* ss often disseminates to synovial tissue during infection which can lead to Lyme arthritis [53]. Previous work by Lemieux et al. [12] and others [54] suggested that OspC type A strains are more associated with dissemination and are linked to more severe symptoms of LB. We did not identify any strain from the internal Dutch collection with the OspC type A sequence. This is likely because OspC type A is restricted within *B. burgdorferi* ss, which represented 3% of the total Dutch isolates. Nevertheless, we observed moderate correlation of the OspC variants and disease stages. For instance, *B. afzelii* OspC variants 17, 19, 55, 56, and 61 were observed in *Borrelia* collected from patients exhibiting EM and ACA. This is consistent with previous clinical results associating *B. afzelii* with skin manifestations [52], and OspC variants from the other three genospecies were not found in conjunction with ACA. In *B. bavariensis*, OspC variant 53, exclusive to this genospecies, was observed in patients exhibiting four different disease stages: EM, EM plus LNB, EM plus Lyme carditis, and LNB. Similarly, variant 60, which was found exclusively in *B. garinii*, was observed in patients with EM plus LNB.

Access to this large WGS collection of clinical Dutch *Borrelia* isolates allowed us to gain a comprehensive picture of OspA diversity among and within *Borrelia* genospecies responsible for LB infections in the Netherlands [35], which is informative to the development of VLA15, a hexavalent OspA-based LB vaccine candidate [55, 56] currently in clinical development [57, 58, 59, 60, 61]. The Dutch *B. afzelii* isolates sequenced in this study all belong to OspA IST2 [35], with the dominant variant (variant 7) serving as the template for the serotype 2 component of VLA15 [56]. As 82% of Dutch *B. afzelii* carried OspA variant 7, as did as 100% of the public *B. afzelii* genomes, VLA15 should provide wide-reaching coverage for this genospecies. Similarly, all Dutch *B. burgdorferi* ss and *B. bavariensis* isolates belong to OspA IST1 and IST4, respectively. Their OspA sequences match the serotype 1 and 4 components included in VLA15. Lastly, the *B. garinii* isolates from the Dutch collection were typed as either OspA IST5 or IST6, the OspA sequences of which were previously shown to exhibit over 95% identity [35] to the serotype 5 and serotype 6 components of VLA15. Taken together, these data indicate that the vast majority of Dutch *Borrelia* in this study are expected to be covered by VLA15 based on amino acid sequence conservation. Wider vaccine coverage could also be expected throughout Europe, as the vast majority (*n* = 36, 92.3%) of this study’s publicly sourced *Borrelia* genomes sampled from human isolates in European countries (Germany, Denmark, Austria, Italy, Slovenia, Sweden, and the Netherlands) encompassed OspA ISTs 1–6, all covered by antigenic components of VLA15.

Limitations of the study include that *Borrelia* clinical isolates were sourced from a single hospital in Amsterdam and, therefore, might not be representative of the complete *Borrelia* sequence diversity from all regions of the Netherlands, although the Amsterdam UMC is a national expertise and referral center for Lyme borreliosis. In addition, isolates collected in this study were not prevalence-based and may not be reflective of the frequency of disease-causing isolates currently circulating in the Netherlands.

## Conclusions

In conclusion, our analysis supports a high genetic diversity observed in *Borrelia* from human cases in the Netherlands, both between and within genospecies, consistent with a prior report comprising many of the Dutch isolates described here [18]. While MLST and IGS retain some predictive value for human infectivity, invasiveness, and clinical LB manifestation, these methods may be less informative to estimating therapeutic or vaccine success. High resolution of the OspA antigen allele, made possible with WGS, revealed that isolates from Dutch human cases were limited to a few OspA serotypes, all of which are included in a hexavalent OspA-based LB vaccine candidate currently undergoing phase 3 clinical trials. The methods utilized in the present study can be expanded to other countries and regions with high rates of LB, should the data be collected and made available. It will prove critical to monitor the evolution of *Borrelia* in regions where a human LB vaccine is widely implemented, as the vaccine may exert pressures that could alter the frequency of certain genospecies, sequence types, or serotypes, potentially affecting coverage.

## Supplementary Information

Supplementary Material 1.

## Data Availability

Raw data presented in this study has been deposited at NCBI SRA under accession PRJNA1041728. Any additional information required to reanalyze the data reported in this publication is available from the corresponding author on reasonable request. Due to data confidentiality reasons, individual patient data cannot be made available.
