# Supplementary material for: Genomic characterization of clinical Borrelia burgdorferi sensu lato isolates in the Netherlands over a thirty-year period
Source: BMC Genomics. 2025 Nov 27;26:1153. doi: 10.1186/s12864-025-12357-4 (PMC12752289; doi:10.1186/s12864-025-12357-4)
Supplement: Supplementary file 1 — Supplementary Material 1. [file 12864_2025_12357_MOESM1_ESM.pdf]

Supplementary Information for

Genomic Characterization of clinical *Borrelia burgdorferi* sensu lato isolates in the Netherlands over a thirty-year period

Zhenghui Li, Jonathan T. Lee, Varun Raghuraman, Lorna D. Nunez, Urvi Rajyaguru, Amber Vrijlandt, Katrina E. Llamera, Lubomira Andrew, Alje P. Van Dam, Annaliesa S. Anderson, Paul A. Liberator, Li Hao, Raphael Simon, Joppe W. Hovius

Contents

Figure S1. Phylogenetic tree of *B. burgdorferi* ss isolates. 2

Figure S2. Phylogenetic tree of *B. garinii* isolates. 3

Figure S3. Phylogenetic tree of *B. bavariensis* isolates. 4

Table S1. Sample and NGS metadata of internally sequenced *Borrelia* isolates. 5

Table S2. Sample and NGS metadata of publicly obtained *Borrelia* isolates. 11

Table S3. Pairwise amino acid sequence identities of seven immune-evasion genes in Dutch *Borrelia* isolates belonging to OspA IST1, IST2, and IST4-6. 18

References 19

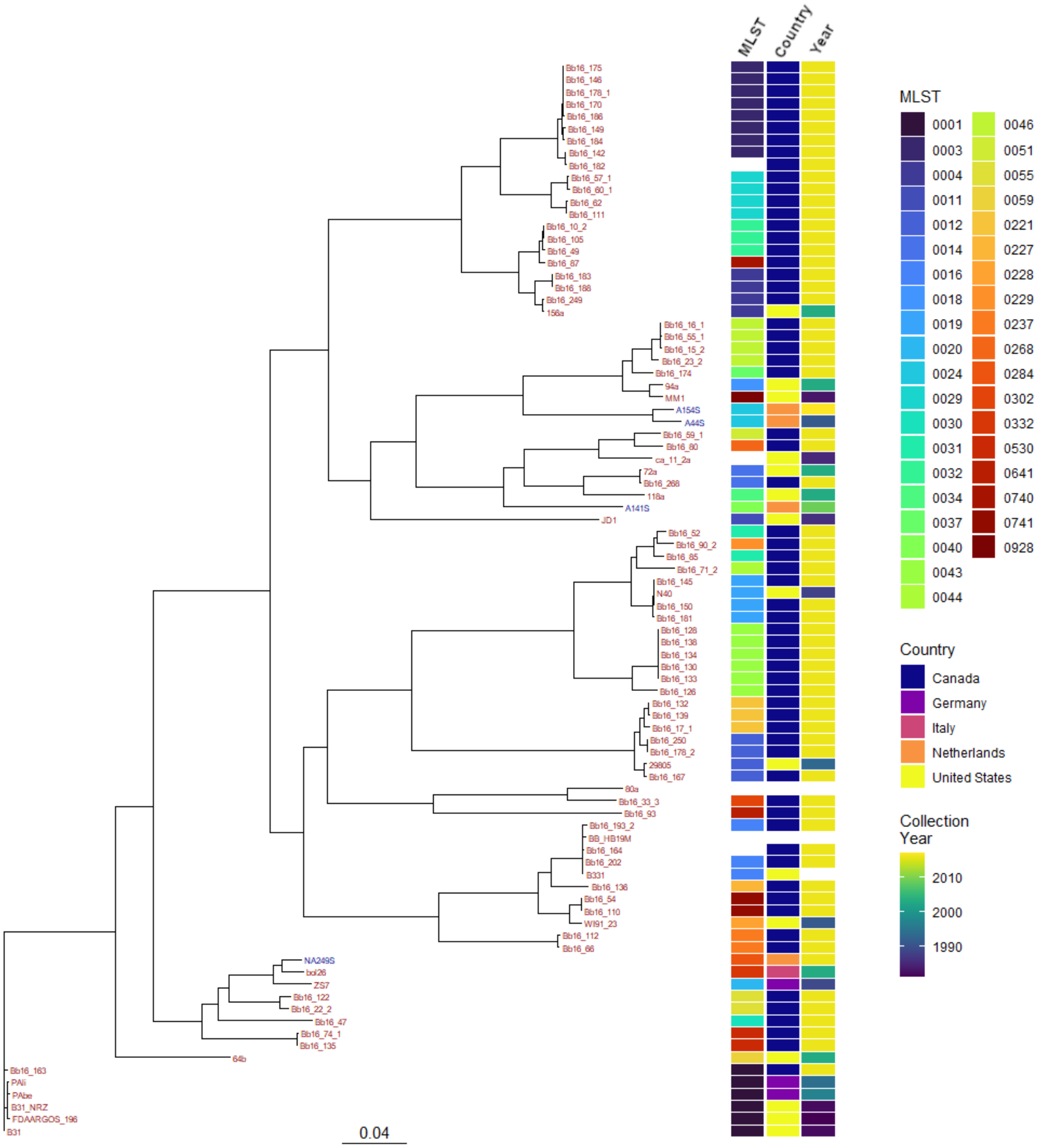

Figure S1. Phylogenetic tree of *B. burgdorferi* ss isolates. Phylogeny and metadata are shown for both the Dutch isolate collection (blue, n =4) and publicly available genomes of *B. burgdorferi* ss (red, n=84). Isolate MLST, country of collection, and collection year are color coded according to their respective color keys. Blank spaces indicate missing metadata or an incomplete MLST.

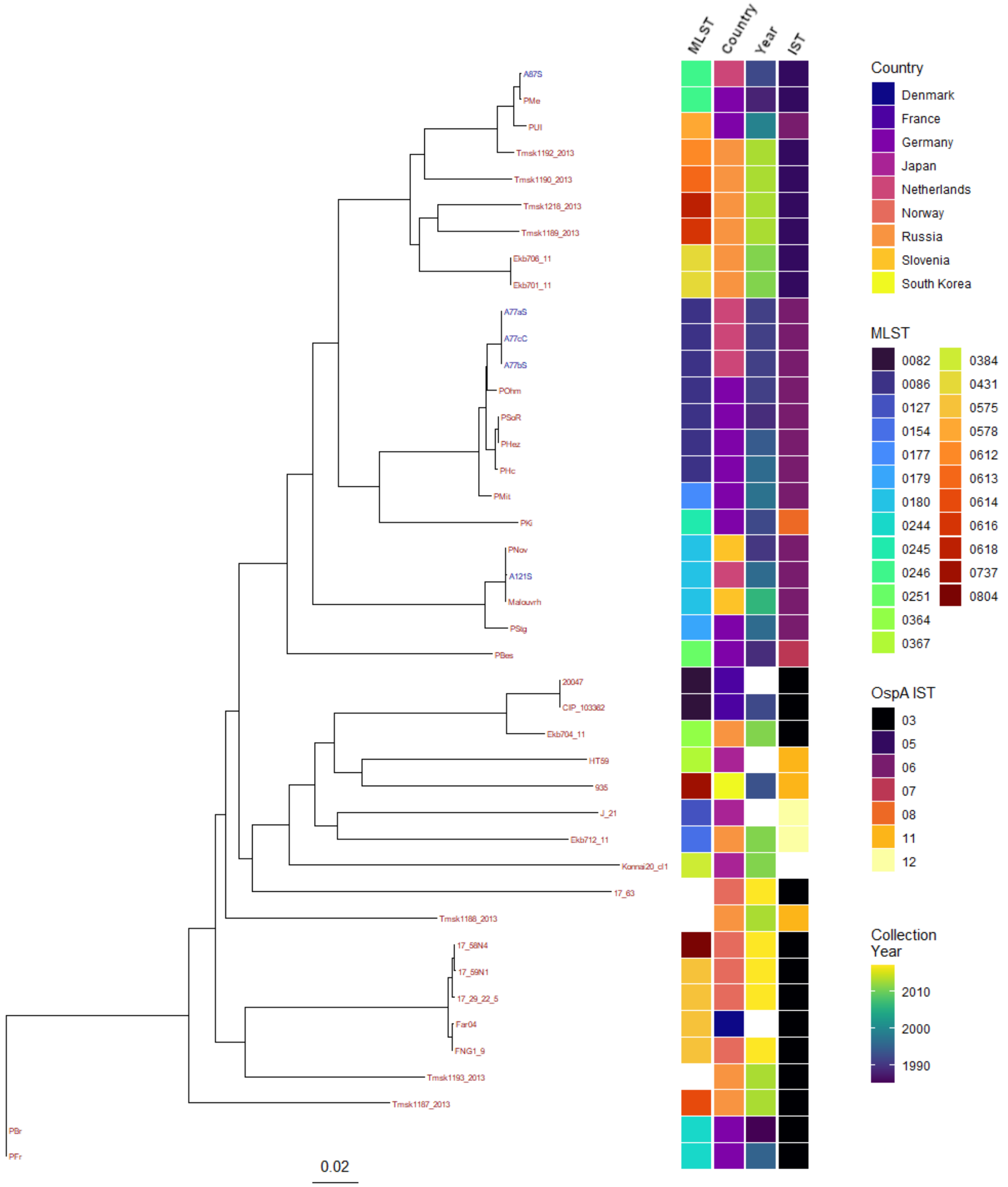

Figure S2. Phylogenetic tree of *B. garinii* isolates. Phylogeny and metadata are shown for both the Dutch isolate collection (blue, n =5) and publicly available genomes of *B. garinii* (red, n=37). Isolate MLST, OspA IST, country of collection, and collection year are color coded according to their respective color keys. Blank spaces indicate missing metadata or an incomplete MLST.

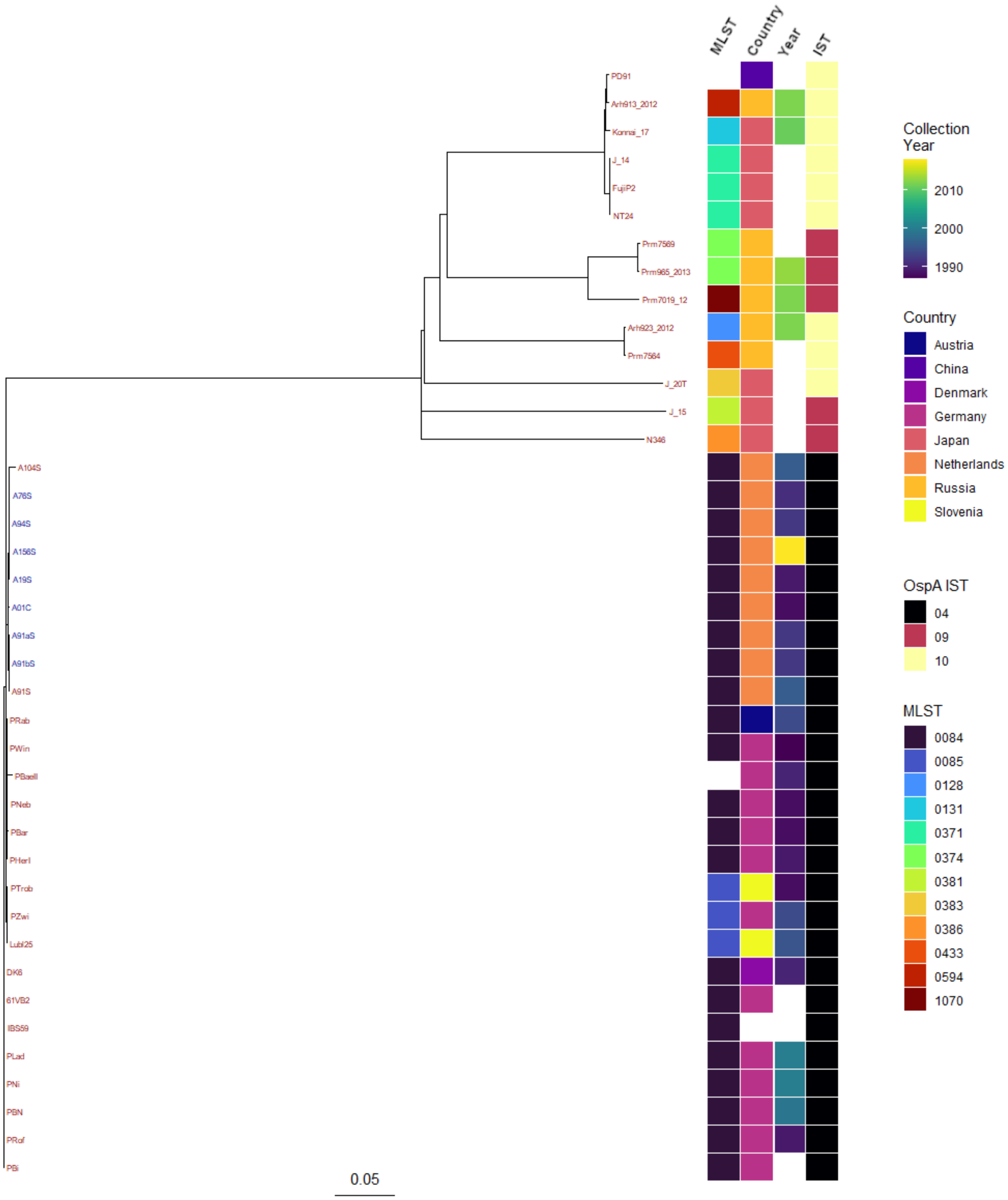

Figure S3. Phylogenetic tree of *B. bavariensis* isolates. Phylogeny and metadata are shown for both the Dutch isolate collection (blue, n =7) and publicly available genomes of *B. bavariensis* (red, n=33). Isolate MLST, OspA IST, country of collection, and collection year are color coded according to their respective color keys. Blank spaces indicate missing metadata or an incomplete MLST.

Table S1. Sample and NGS metadata of internally sequenced *Borrelia* isolates.

Isolate	PFE IDs	Accession	Species	Source	Disease Stage	Year of Collection	*clpA*	*clpX*	*nifS*	*pepX*	*pyrG*	*recG*	*rplB*	*uvrA*	MLST	Coipan et al. 2016[a]	OspA Variant	OspA IST	OspC Variant	DbpA Variant	Chromosomal Coverage
A01C	PFELB0071	SAMN38696040	*B. bavariensis*	CSF	LNB	1988	41	26	27	37	28	35	26	32	84	mismatch	12	4	53	43	113
A02S	PFELB0072	SAMN38696041	*B. afzelii*	skin	EM	1989	36	24	24	277	22	29	23	28	1039	NA	7	2	17	16	388
A03S	PFELB0073	SAMN38696042	*B. afzelii*	skin	EM	1989	36	24	24	277	22	29	23	28	1039	NA	7	2	17	16	35
A04S	PFELB0074	SAMN38696043	*B. afzelii*	skin	EM	1989	36	24	24	277	22	29	23	28	1039	NA	7	2	17	16	416
A05S	PFELB0075	SAMN38696044	*B. afzelii*	skin	EM	1989	109	24	23	89	96	27	23	28	467	NA	7	2	56	63	411
A06S	PFELB0076	SAMN38696045	*B. afzelii*	skin	EM	1989	36	24	24	277	22	29	23	28	1039	NA	7	2	17	16	326
A07S	PFELB0077	SAMN38696046	*B. afzelii*	skin	EM	1989	36	24	119	32	20	27	183	28	710	NA	7	2	19	64	407
A08S	PFELB0078	SAMN38696047	*B. afzelii*	skin	EM	1989	114	24	24	31	22	92	23	28	347	NA	61	2	54	16	280
A09S	PFELB0079	SAMN38696048	*B. afzelii*	skin	ACA	1989	36	24	24	277	22	29	23	28	1039	mismatch	7	2	17	16	429
A100S	PFELB0144	SAMN38698425	*B. afzelii*	skin	ACA	1993	109	24	23	31	23	30	23	28	753	753	65	2	61	81	304
A101S	PFELB0145	SAMN38698426	*B. afzelii*	skin	EM	1993	109	24	24	85	22	91	24	29	476	476	61	2	55	83	263
A102S	PFELB0146	SAMN38698427	*B. afzelii*	skin	EM	1993	36	24	23	86	22	27	23	28	165	165	7	2	56	83	254
A103S	PFELB0147	SAMN38698428	*B. afzelii*	skin	EM	1993	37	24	23	31	22	29	23	28	289	289	7	2	17	16	279
A105S	PFELB0148	SAMN38698429	*B. afzelii*	skin	EM	1993	36	24	119	32	20	27	183	28	710	SLV of 710	7	2	19	64	127
A106S	PFELB0001	SAMN38695972	*B. afzelii*	skin	EM	1993	109	24	24	49	25	27	23	28	249	SLV of 249	7	2	65	15	157
A107S	PFELB0149	SAMN38698430	*B. afzelii*	skin	EM	1993	109	24	23	89	96	27	23	28	467	467	7	2	56	63	172
A108S	PFELB0150	SAMN38698431	*B. afzelii*	skin	EM	1993	36	24	24	31	22	29	23	28	1034	1034	7	2	17	68	231
A109S	PFELB0151	SAMN38698432	*B. afzelii*	skin	EM	1994	109	24	24	85	22	91	24	29	476	476	61	2	55	63	189
A10S	PFELB0080	SAMN38696049	*B. afzelii*	skin	EM	1989	36	24	24	31	22	29	23	28	1034	NA	7	2	17	16	405
A110S	PFELB0152	SAMN38698433	*B. afzelii*	skin	ACA	1994	35	23	22	32	20	27	23	28	354	354	7	2	72	89	271
A111aS	PFELB0153	SAMN38698434	*B. afzelii*	skin	ACA	1994	36	24	23	32	20	27	183	28	710	mismatch	61	2	19	64	323
A111bS	PFELB0154	SAMN38698435	*B. afzelii*	skin	ACA	1994	36	24	23	32	20	27	183	28	710	710	61	2	19	64	213
A112S	PFELB0155	SAMN38698380	*B. afzelii*	skin	EM	1994	109	24	24	85	22	91	24	29	476	476	61	2	55	63	216
A113S	PFELB0156	SAMN38698381	*B. afzelii*	skin	EM	1994	36	24	23	86	22	27	23	28	165	165	7	2	56	83	169

Isolate	PFE IDs	Accession	Species	Source	Disease Stage	Year of Collection	*clpA*	*clpX*	*nifS*	*pepX*	*pyrG*	*recG*	*rplB*	*uvrA*	MLST	Coipan et al. 2016[a]	OspA Variant	OspA IST	OspC Variant	DbpA Variant	Chromosomal Coverage
A114S	PFELB0157	SAMN38698382	*B. afzelii*	skin	ACA	1994	109	24	25	86	107	29	23	285	327	SLV of 327	7	2	64	75	221
A115S	PFELB0158	SAMN38698383	*B. afzelii*	skin	EM	1994	36	24	24	277	22	29	23	28	1039	1039	7	2	17	16	229
A116S	PFELB0159	SAMN38698384	*B. afzelii*	skin	EM	1995	36	24	119	32	20	27	183	28	710	SLV of 710	7	2	19	64	181
A117S	PFELB0160	SAMN38698385	*B. afzelii*	skin	EM	1995	39	24	24	31	22	92	23	28	171	171	7	2	17	16	43
A118S	PFELB0161	SAMN38698386	*B. afzelii*	skin	EM	1995	37	24	23	31	22	29	23	28	289	mismatch	7	2	17	16; 67	116
A119S	PFELB0162	SAMN38698387	*B. afzelii*	skin	EM	1996	109	24	24	49	25	27	23	28	249	SLV of 249	7	2	65	15	212
A11S	PFELB0081	SAMN38696050	*B. afzelii*	skin	ACA	1989	36	24	24	31	22	29	23	28	1034	NA	7	2	17	68	161
A120S	PFELB0163	SAMN38698388	*B. afzelii*	skin	EM	1996	36	24	23	32	20	27	183	28	710	710	61	2	19	64	154
A121S	PFELB0164	SAMN38698389	*B. garinii*	skin	EM	1996	43	28	30	90	87	36	28	34	180	180	21	6	36	27	199
A122S	PFELB0165	SAMN38698390	*B. afzelii*	skin	EM	1996	109	24	24	286	22	29	23	28	256	mismatch	7	2	17	16	278
A123S	PFELB0166	SAMN38698391	*B. afzelii*	skin	EM	1997	109	24	253	89	96	27	23	28	467	SLV of 467	7	2	56	87	160
A126S	PFELB0167	SAMN38698392	*B. afzelii*	skin	EM	1997	35	24	23	89	96	27	23	28	467	SLV of 467	7	2	56	63	254
A127S	PFELB0168	SAMN38698393	*B. afzelii*	skin	EM	1998	N	24	N	32	90	29	24	28	463	DLV of 463	7	2	66	76	160
A128S	PFELB0169	SAMN38698394	*B. afzelii*	skin	EM	1999	109	24	24	85	22	91	24	29	476	untype	61	2	55	65	272
A129S	PFELB0170	SAMN38698395	*B. afzelii*	skin	EM	1999	36	24	24	277	22	29	23	28	1039	untype	7	2	17	16	250
A12S	PFELB0082	SAMN38696051	*B. afzelii*	skin	EM	1989	35	24	24	30	21	29	23	28	72	NA	74	2	20	62	262
A130S	PFELB0171	SAMN38698396	*B. afzelii*	skin	ACA	2003	37	24	23	31	22	29	23	28	289	untype	7	2	17	16	88
A131S	PFELB0172	SAMN38698397	*B. afzelii*	skin	ACA	2004	38	24	25	32	90	239	24	28	781	untype	7	2	78	77	117
A132S	PFELB0173	SAMN38698398	*B. afzelii*	skin	ACA	2004	36	24	23	87	92	27	23	78	846	untype	7	2	63	74	84
A133S	PFELB0174	SAMN38698399	*B. afzelii*	skin	EM	2006	36	24	24	89	92	27	23	286	166	untype	7	2	56	63	113
A134S	PFELB0175	SAMN38698400	*B. afzelii*	skin	EM	2006	51	24	24	31	90	27	23	30	731	untype	7	2	17	85	162
A135S	PFELB0176	SAMN38698401	*B. afzelii*	skin	EM	2006	36	24	24	277	22	29	23	28	1039	untype	7	2	17	16	176
A136S	PFELB0177	SAMN38698402	*B. afzelii*	skin	ACA	2006	36	24	24	277	-	-	23	28	1039	untype	7	2	17	16	113
A138S	PFELB0178	SAMN38698403	*B. afzelii*	skin	EM	2008	36	24	23	31	23	30	23	30	75	untype	7	2	61	P	113
A13S	PFELB0083	SAMN38696052	*B. afzelii*	skin	EM	1989	109	24	24	85	22	91	24	29	476	NA	61	2	55	63	203
A140S	PFELB0179	SAMN38698404	*B. afzelii*	skin	EM	2009	36	24	24	277	22	29	23	28	1039	NA	7	2	17	16	122

Isolate	PFE IDs	Accession	Species	Source	Disease Stage	Year of Collection	*clpA*	*clpX*	*nifS*	*pepX*	*pyrG*	*recG*	*rplB*	*uvrA*	MLST	Coipan et al. 2016[a]	OspA Variant	OspA IST	OspC Variant	DbpA Variant	Chromosomal Coverage
A141S	PFELB0180	SAMN38698405	*B. burgdorferi*	skin	MEM	2009	22	1	5	8	1	18	11	10	40	NA	3	1	67	78	157
A142S	PFELB0181	SAMN38698406	*B. afzelii*	skin	EM	2009	37	24	24	31	22	29	23	29	73	NA	7	2	17	16	84
A149S	PFELB0182	SAMN38698407	*B. afzelii*	skin	ACA	2013	39	24	23	32	96	165	23	156	697	NA	7	2	68	15	159
A14S	PFELB0084	SAMN38696053	*B. spielmanii*	skin	EM	1989	94	72	71	84	80	75	72	76	159	159	51	13	71	86	40
A150S	PFELB0183	SAMN38698408	*B. afzelii*	skin	EM	2014	36	24	23	31	85	27	23	29	168	NA	7	2	19	79	193
A151S	PFELB0184	SAMN47848232	*B. afzelii*	skin	ACA	2014	109	24	24	85	22	91	24	29	476	mismatch	61	2	55	65	94
A152S	PFELB0185	SAMN38698409	*B. afzelii*	skin	EM	2016	36	24	24	277	22	29	23	28	1039	NA	7	2	17	16	170
A153S	PFELB0186	SAMN38698410	*B. afzelii*	skin	ACA	2017	109	24	24	85	22	91	24	29	476	NA	61	2	55	65	123
A154S	PFELB0187	SAMN38698411	*B. burgdorferi*	skin	EM	2017	N	9	12	8	1	11	8	16	24	NA	1	1	P	P	169
A156S	PFELB0188	SAMN38698412	*B. bavariensis*	skin	EM + LC	2018	41	26	27	37	28	35	26	32	84	NA	12	4	53	43	59
A157S	PFELB0189	SAMN38698413	*B. afzelii*	skin	EM	2018	37	24	24	31	22	29	23	28	263	NA	7	2	17	16	119
A159S	PFELB0190	SAMN38698414	*B. afzelii*	skin	EM	2018	36	24	23	31	23	N	23	30	75	NA	7	2	61	63	206
A15S	PFELB0085	SAMN38696054	*B. afzelii*	skin	EM	1989	38	24	25	32	163	29	252	28	474	SLV of 474	7	2	16	88	253
A161S	PFELB0207	SAMN47848227	*B. afzelii*	skin	EM	2021	51	24	23	86	85	92	23	29	817	NA	7	2	20	63	63
A162S	PFELB0208	SAMN47848228	*B. afzelii*	skin	ACA	2021	36	24	23	86	22	27	23	28	165	NA	7	2	56	83	71
A163S	PFELB0209	SAMN47848229	*B. afzelii*	skin	EM	2021	36	24	24	277	22	29	23	28	1039	NA	7	2	17	16	155
A164S	PFELB0210	SAMN47848230	*B. afzelii*	skin	EM	2022	36	24	25	31	272	92	23	29	U	NA	7	2	61	84	98
A165S	PFELB0211	SAMN47848231	*B. afzelii*	skin	MEM	2023	36	23	22	285	26	96	23	156	U	NA	7	2	58	66	48
A16S	PFELB0086	SAMN38696055	*B. afzelii*	skin	ACA	1989	36	24	N	32	20	27	183	28	710	SLV of 710	7	2	19	63	390
A17S	PFELB0087	SAMN38696056	*B. afzelii*	skin	ACA	1989	36	24	23	31	23	30	23	30	75	mismatch	7	2	61	16	196
A18S	PFELB0088	SAMN38696057	*B. afzelii*	skin	EM	1989	109	24	23	89	96	27	23	28	467	467	7	2	56	64	272
A19S	PFELB0089	SAMN38696058	*B. bavariensis*	skin	EM + LNB	1989	41	26	27	37	28	35	26	32	84	84	12	4	53	43	16
A20S	PFELB0090	SAMN38696059	*B. afzelii*	skin	EM	1990	109	24	23	89	96	27	23	28	467	467	7	2	56	63	219
A21S	PFELB0091	SAMN38696060	*B. afzelii*	skin	EM	1990	36	24	24	277	22	29	23	28	1039	1039	7	2	17	16	216
A22S	PFELB0092	SAMN38696061	*B. afzelii*	skin	EM	1990	36	24	24	277	22	29	23	28	1039	1039	7	2	17	16	426

Isolate	PFE IDs	Accession	Species	Source	Disease Stage	Year of Collection	*clpA*	*clpX*	*nifS*	*pepX*	*pyrG*	*recG*	*rplB*	*uvrA*	MLST	Coipan et al. 2016[a]	OspA Variant	OspA IST	OspC Variant	DbpA Variant	Chromosomal Coverage
A23S	PFELB0093	SAMN38696062	*B. afzelii*	skin	EM	1990	39	24	24	31	22	92	23	28	171	untype	7	2	17	16	272
A24S	PFELB0094	SAMN38696063	*B. afzelii*	skin	EM	1990	36	24	23	31	85	27	23	29	168	mismatch	7	2	19	79	53
A26S	PFELB0096	SAMN38696064	*B. afzelii*	skin	ACA + LA	1990	36	24	23	31	20	30	23	30	75	SLV of 75	61	2	61	72	223
A27S	PFELB0097	SAMN38696065	*B. afzelii*	skin	EM	1990	36	24	25	31	272	92	23	29	676	DLV of 676	7	2	61	68	191
A28S	PFELB0098	SAMN38696066	*B. afzelii*	skin	EM	1990	39	24	24	31	22	92	23	28	171	171	7	2	17	16	102
A29S	PFELB0099	SAMN38696067	*B. afzelii*	skin	EM	1990	109	24	253	89	96	27	23	28	467	NA	7	2	56	87	111
A30S	PFELB0100	SAMN38696068	*B. afzelii*	skin	EM	1990	36	24	24	31	22	29	23	28	76	1039	7	2	17	16	261
A31S	PFELB0101	SAMN38696069	*B. afzelii*	skin	EM	1990	36	24	24	31	96	27	23	28	790	SLV of 790	7	2	59	69	168
A32S	PFELB0102	SAMN38696070	*B. afzelii*	skin	EM	1990	114	24	24	31	22	92	23	28	347	347	61	2	17	16	114
A33S	PFELB0103	SAMN38696071	*B. afzelii*	skin	EM	1990	109	24	24	85	119	27	24	29	783	783	7	2	55	70	248
A34S	PFELB0104	SAMN38696072	*B. afzelii*	skin	EM	1990	109	24	24	85	22	91	24	29	476	mismatch	61	2	55	65	303
A35S	PFELB0105	SAMN38696073	*B. afzelii*	skin	EM	1990	39	24	24	31	22	92	23	28	171	171	7	2	17	16	39
A36S	PFELB0106	SAMN38696074	*B. afzelii*	skin	EM	1990	109	24	24	85	22	91	24	29	476	476	61	2	55	65	97
A37S	PFELB0107	SAMN38696075	*B. afzelii*	skin	EM	1990	37	24	23	31	22	29	23	28	289	289	7	2	17	16	110
A38S	PFELB0108	SAMN38696076	*B. afzelii*	skin	EM	1990	36	23	22	285	26	96	23	156	716	DLV of 716	7	2	58	66	151
A39S	PFELB0109	SAMN38696077	*B. afzelii*	skin	EM	1990	109	24	24	85	22	91	24	29	476	476	61	2	55	65	21
A40S	PFELB0110	SAMN38696078	*B. afzelii*	skin	EM	1991	51	24	23	86	85	92	23	29	817	NA	7	2	20	67	170
A41S	PFELB0111	SAMN38696079	*B. afzelii*	skin	EM	1991	36	24	24	277	22	29	23	28	1039	1039	7	2	17	16	142
A42S	PFELB0112	SAMN38696080	*B. afzelii*	skin	EM	1991	37	24	23	31	22	92	23	28	170	SLV of 170	7	2	17	16	207
A43S	PFELB0113	SAMN38696081	*B. afzelii*	skin	EM	1991	109	24	24	85	22	91	24	29	476	476	61	2	55	65	207
A44S	PFELB0114	SAMN38696082	*B. burgdorferi*	skin	EM	1991	15	287	12	8	1	11	8	16	24	SLV of 24	1	1	62	73	139
A45aS	PFELB0115	SAMN38696083	*B. afzelii*	skin	EM	1991	36	23	22	285	26	96	23	156	716	untype	7	2	58	66	174
A45bS	PFELB0116	SAMN38696084	*B. afzelii*	skin	EM	1991	36	23	22	285	26	96	23	156	716	NA	7	2	58	66	150
A47S	PFELB0117	SAMN38696085	*B. afzelii*	skin	EM	1991	36	24	23	31	96	27	23	28	561	SLV of 561	7	2	59	63	184
A74S	PFELB0118	SAMN38696086	*B. afzelii*	skin	EM	1991	36	24	119	32	20	27	183	28	710	SLV of 710	7	2	19	64	232
A76S	PFELB0119	SAMN38696087	*B. bavariensis*	skin	EM + LC	1991	41	26	27	37	28	35	26	32	84	84	12	4	53	43	13

Isolate	PFE IDs	Accession	Species	Source	Disease Stage	Year of Collection	*clpA*	*clpX*	*nifS*	*pepX*	*pyrG*	*recG*	*rplB*	*uvrA*	MLST	Coipan et al. 2016[a]	OspA Variant	OspA IST	OspC Variant	DbpA Variant	Chromosomal Coverage
A77aS	PFELB0120	SAMN38696088	*B. garinii*	skin	EM + LNB	1991	42	27	29	38	29	36	27	33	86	86	18	6	60	71	475
A77bS	PFELB0121	SAMN38696089	*B. garinii*	skin	EM + LNB	1991	42	27	29	38	29	36	27	33	86	86	18	6	60	71	383
A77cC	PFELB0122	SAMN38696090	*B. garinii*	CSF	EM + LNB	1991	42	27	29	38	29	36	27	33	86	86	18	6	60	71	258
A81S	PFELB0124	SAMN38696092	*B. afzelii*	skin	EM	1991	36	24	24	277	22	29	23	28	1039	NA	7	2	17	16	19
A82S	PFELB0125	SAMN38696093	*B. afzelii*	skin	EM	1992	38	24	25	32	163	29	252	28	474	NA	7	2	16	88	108
A83S	PFELB0126	SAMN38696094	*B. afzelii*	skin	EM	1992	51	24	23	86	85	27	23	28	349	NA	7	2	20	67	167
A84S	PFELB0127	SAMN38696095	*B. afzelii*	skin	EM	1992	36	24	24	277	22	29	23	28	1039	1039	7	2	17	16	251
A85S	PFELB0128	SAMN38696096	*B. afzelii*	skin	EM	1992	37	24	24	31	85	92	23	28	170	SLV of 170	7	2	17	64	334
A86S	PFELB0129	SAMN38696097	*B. afzelii*	skin	ACA	1992	37	24	24	31	22	29	23	28	263	263	7	2	17	16	230
A87S	PFELB0130	SAMN38696098	*B. garinii*	skin	EM + LNB	1992	112	80	78	99	81	39	79	87	246	246	14	5	32	31	229
A88S	PFELB0131	SAMN38696099	*B. afzelii*	skin	ACA	1992	39	24	24	31	22	92	23	28	171	171	7	2	P	16	131
A89S	PFELB0132	SAMN38696100	*B. afzelii*	skin	EM	1992	109	24	23	89	96	27	23	28	467	467	7	2	56	63	184
A90S	PFELB0133	SAMN38696101	*B. afzelii*	skin	EM	1992	IC	24	23	86	85	92	23	29	817	817	7	2	20	63	22
A91aS	PFELB0134	SAMN38696102	*B. bavariensis*	skin	EM + LNB	1992	41	26	27	37	28	35	26	32	84	untype	12	4	53	43	105
A91bC	PFELB0135	SAMN38696103	*B. bavariensis*	CSF	EM + LNB	1992	41	26	27	37	28	35	26	32	84	84	12	4	53	43	14
A92S	PFELB0136	SAMN38696104	*B. afzelii*	skin	EM	1992	39	24	24	31	22	92	23	28	171	171	7	2	17	16	143
A93S	PFELB0137	SAMN38696105	*B. afzelii*	skin	EM	1992	109	145	23	31	23	30	23	29	473	SLV of 473	65	2	61	64	213
A94S	PFELB0138	SAMN38696106	*B. bavariensis*	skin	EM	1992	41	26	27	37	28	35	26	32	84	84	12	4	53	43	165
A95S	PFELB0139	SAMN38696107	*B. afzelii*	skin	ACA	1992	36	24	23	87	92	27	23	78	846	846	7	2	63	74	216
A96S	PFELB0140	SAMN38696108	*B. afzelii*	skin	EM	1992	36	24	24	277	22	29	23	28	1039	1039	7	2	17	16	217
A97S	PFELB0141	SAMN38698423	*B. afzelii*	skin	EM	1992	36	24	24	277	22	29	23	28	1039	mismatch	7	2	17	16	253
A99S	PFELB0143	SAMN38698424	*B. afzelii*	skin	EM	1993	36	24	119	32	20	27	271	28	710	DLV of 710	7	2	19	64	261
NA112S	PFELB0191	SAMN38698415	*B. afzelii*	skin	EM	2015	109	24	24	286	22	29	23	28	256	NA	7	2	17	16	199

Isolate	PFE IDs	Accession	Species	Source	Disease Stage	Year of Collection	*clpA*	*clpX*	*nifS*	*pepX*	*pyrG*	*recG*	*rplB*	*uvrA*	MLST	Coipan et al. 2016[a]	OspA Variant	OspA IST	OspC Variant	DbpA Variant	Chromosomal Coverage
NA209 S	PFELB0192	SAMN38698416	*B. afzelii*	skin	EM	2016	109	24	23	89	96	27	23	28	467	NA	7	2	56	63	78
NA246 S	PFELB0193	SAMN38698417	*B. afzelii*	skin	EM	2016	36	24	23	31	23	30	23	30	75	NA	7	2	61	81	120
NA249 S	PFELB0194	SAMN38698418	*B. burgdorferi*	skin	ACA + LNB	2016	14	1	11	1	1	11	1	10	284	NA	3	1	69	82	192
NA384 S	PFELB0195	SAMN38698419	*B. afzelii*	skin	EM	2017	109	24	24	85	22	91	24	29	476	NA	61	2	55	65	120
NA392 S	PFELB0196	SAMN38698420	*B. afzelii*	skin	EM	2017	36	24	23	86	22	27	23	28	165	NA	7	2	56	83	165
NA503 S	PFELB0197	SAMN38698421	*B. afzelii*	skin	EM	2018	36	24	25	32	272	92	23	29	78	NA	7	2	61	84	262

[a] MLST of given isolate assigned in Coipan et al. [1]

Abbreviations: CSF, cerebral spinal fluid; EM, erythema migrans; MEM, multiple erythema migrans; LA, Lyme arthritis; LC, Lyme carditis; LNB, Lyme neuroborreliosis; ACA, acrodermatitis chronica atrophicans; IC, incomplete; MLST, multilocus sequence type; DLV, double-locus variant; SLV, single-locus variant; IST, *in silico* type; U, unknown; NA, not applicable; P, partial; N, novel

Table S2. Sample and NGS metadata of publicly obtained *Borrelia* isolates.

Isolate	PubMLST ID	Species	Source	Country	Year of Collection	*clpA*	*clpX*	*nifS*	*pepX*	*pyrG*	*recG*	*rplB*	*uvrA*	ST (MLST)	*ospA* Allele	OspA Variant	OspA IST	OspC Variant	*dbpA*	DbpA Variant
B31	1	*B. burgdorferi*	tick	United States	1982	1	1	1	1	1	1	1	1	1	1	1	1	1	1	1
PAli	U	*B. burgdorferi*	human	Germany	1994	1	1	1	1	1	1	1	1	1	1	1	1	1	1	1
PAbe	U	*B. burgdorferi*	human	Germany	1997	1	1	1	1	1	1	1	1	1	1	1	1	1	1	1
FDAARGOS_196	U	*B. burgdorferi*	tick	United States	1981	1	1	1	1	1	1	1	1	1	1	1	1	1	1	1
Bb16-163	U	*B. burgdorferi*	tick	Canada	2016	1	1	1	1	1	1	1	1	1	1	1	1	1	1	1
Bb16-122	U	*B. burgdorferi*	tick	Canada	2016	23	1	17	20	2	1	1	10	55	7	4	1	1	U	1
Bb16-22-2	U	*B. burgdorferi*	tick	Canada	2016	23	1	17	20	2	1	1	10	55	96	4	1	1	U	1
B31_NRZ	U	*B. burgdorferi*	tick	United States	1982	1	1	1	1	1	1	1	1	1	1	1	1	1	1	1
ca_11_2a	U	*B. burgdorferi*	tick pacificus	United States	1985	8	1	U	120	1	18	1	7	U	9	6	1	2	7	7
Bb16-59-1	U	*B. burgdorferi*	tick	Canada	2016	8	1	15	8	12	20	1	7	51	3	2	1	2	U	U
Bb16-80	U	*B. burgdorferi*	tick	Canada	2016	8	1	15	94	2	20	96	7	268	4	3	1	2	7	7
JD1	16	*B. burgdorferi*	tick	United States	1986	5	7	5	1	6	1	4	9	11	7	4	1	3	4	4
64b	1067	*B. burgdorferi*	human	United States	2003	6	1	5	1	1	7	1	19	59	3	2	1	4	10	10
WI91_23	U	*B. burgdorferi*	bird	United States	1991	12	2	1	2	1	16	2	2	228	4	3	1	5	12	12
B331	3	*B. burgdorferi*	human	United States	U	2	2	1	2	2	2	2	2	16	54	3	1	5	12	12
Bb16-136	U	*B. burgdorferi*	tick	Canada	2016	12	2	1	2	1	16	76	2	227	78	3	1	5	12	12
Bb16-54	U	*B. burgdorferi*	tick	Canada	2016	244	2	1	2	1	16	4	2	741	96	4	1	5	12	12
Bb16-110	U	*B. burgdorferi*	tick	Canada	2016	244	2	1	2	1	16	4	2	741	96	4	1	5	12	12
Bb16-193-2	U	*B. burgdorferi*	tick	Canada	2016	2	2	1	2	2	2	2	2	16	54	3	1	5	12	12
Bb16-202	U	*B. burgdorferi*	tick	Canada	2016	2	2	1	2	2	2	2	2	16	54	3	1	5	12	12
Bb16-164	U	*B. burgdorferi*	tick	Canada	2016	2	2	U	2	2	2	2	2	U	54	3	1	5	12	12
BB-HB19M	U	*B. burgdorferi*	U	U	U	2	2	U	2	2	2	2	2	U	54	3	1	5	12	12
ZS7	1068	*B. burgdorferi*	tick ricinus	Germany	1989	14	1	11	1	1	1	1	10	20	8	5	1	6	8	8
Bb16-182	U	*B. burgdorferi*	tick	Canada	2016	4	1		1	1	6	1	7	U	2	2	1	7	90	90
Bb16-142	U	*B. burgdorferi*	tick	Canada	2016	4	1	1	1	1	6	1	7	3	2	2	1	7	90	90

Isolate	PubMLST ID	Species	Source	Country	Year of Collection	*clpA*	*clpX*	*nifS*	*pepX*	*pyrG*	*recG*	*rplB*	*uvrA*	ST (MLST)	*ospA* Allele	OspA Variant	OspA IST	OspC Variant	*dbpA*	DbpA Variant
Bb16-184	U	*B. burgdorferi*	tick	Canada	2016	4	1	1	1	1	6	1	7	3	2	2	1	7	2	2
Bb16-149	U	*B. burgdorferi*	tick	Canada	2016	4	1	1	1	1	6	1	7	3	2	2	1	7	2	2
Bb16-175	U	*B. burgdorferi*	tick	Canada	2016	4	1	1	1	1	6	1	7	3	2	2	1	7	2	2
Bb16-170	U	*B. burgdorferi*	tick	Canada	2016	4	1	1	1	1	6	1	7	3	2	2	1	7	2	2
Bb16-146	U	*B. burgdorferi*	tick	Canada	2016	4	1	1	1	1	6	1	7	3	2	2	1	7	2	2
Bb16-178-1	U	*B. burgdorferi*	tick	Canada	2016	4	1	1	1	1	6	1	7	3	2	2	1	7	2	2
Bb16-186	U	*B. burgdorferi*	tick	Canada	2016	4	1	1	1	1	6	1	7	3	2	2	1	7	2	2
118a	1072	*B. burgdorferi*	human	United States	2003	8	1	1	7	1	6	1	10	34	7	4	1	8	6	6
Bb16-112	U	*B. burgdorferi*	tick	Canada	2016	107	2	1	95	2	18	4	3	237	78	3	1	8	U	U
Bb16-66	U	*B. burgdorferi*	tick	Canada	2016	107	2	1	95	2	18	4	3	237	78	3	1	8	U	U
94a	1074	*B. burgdorferi*	human	United States	2003	7	6	6	1	1	5	5	5	18	7	4	1	9	5	5
MM1	U	*B. burgdorferi*	mouse	United States	1983	7	6	236	265	1	5	5	5	928	53	48	1	9	5	5
156a	1075	*B. burgdorferi*	human	United States	2003	8	1	1	1	4	6	1	7	4	2	2	1	10	3	3
Bb16-49	U	*B. burgdorferi*	tick	Canada	2016	8	1	1	1	4	16	1	7	32	4	3	1	10	3	3
Bb16-105	U	*B. burgdorferi*	tick	Canada	2016	8	1	1	1	4	16	1	7	32	4	3	1	10	3	3
Bb16-10-2	U	*B. burgdorferi*	tick	Canada	2016	8	1	1	1	4	16	1	7	32	4	3	1	10	3	3
Bb16-249	U	*B. burgdorferi*	tick	Canada	2016	8	1	1	1	4	6	1	7	4	2	2	1	10	3	3
Bb16-87	U	*B. burgdorferi*	tick	Canada	2016	8	1	1	1	5	6	1	7	740	4	3	1	10	3	3
29805	1073	*B. burgdorferi*	tick	United States	1993	3	3	2	4	3	4	4	4	12	2	2	1	12	9	9
Bb16-167	U	*B. burgdorferi*	tick	Canada	2016	3	3	2	4	3	4	4	4	12	2	2	1	12	9	9
N40	25	*B. burgdorferi*	tick	United States	1988	4	4	3	3	3	3	3	3	19	5	3	1	13	11	11
80a	U	*B. burgdorferi*	U	U	U	U	5	4	6	1	6	1	6	U	55	2	1	13	2;11	2;11
Bb16-181	U	*B. burgdorferi*	tick	Canada	2016	4	4	3	3	3	3	3	3	19	5	3	1	13	11	11
Bb16-145	U	*B. burgdorferi*	tick	Canada	2016	4	4	3	3	3	3	3	3	19	5	3	1	13	11	11
Bb16-150	U	*B. burgdorferi*	tick	Canada	2016	4	4	3	3	3	3	3	3	19	5	3	1	13	11	11
72a	1070	*B. burgdorferi*	human	United States	2003	9	1	1	7	1	6	1	10	14	5	3	1	14	6	6
Bb16-268	U	*B. burgdorferi*	tick	Canada	2016	9	1	1	7	1	6	1	10	14	5	3	1	14	6	6

Isolate	PubMLST ID	Species	Source	Country	Year of Collection	*clpA*	*clpX*	*nifS*	*pepX*	*pyrG*	*recG*	*rplB*	*uvrA*	ST (MLST)	*ospA* Allele	OspA Variant	OspA IST	OspC Variant	*dbpA*	DbpA Variant
bol26	1066	*B. burgdorferi*	human	Italy	2003	14	1	11	119	1	1	1	10	332	6	3	1	15	8	8
K78	U	*B. afzelii*	human	Austria	2014	109	94	24	34	21	29	99	28	335	10	7	2	16	13	13
ACA-1	U	*B. afzelii*	human	Sweden	U	37	24	24	89	22	220	23	28	1071	10	7	2	17	16	16
BO23	U	*B. afzelii*	human	Germany	1995	109	24	23	32	25	32	23	29	698	11	7	2	18	15	15
IPT138	U	*B. afzelii*	tick	France	2004	36	24	U	U	25	32	23	29	U	P	P	U	18	15	15
PKo	139	*B. afzelii*	human	Germany	1984	36	24	23	29	20	28	23	29	71	11	7	2	19	14	14
PBr	U	*B. garinii*	human	Germany	1985	48	34	34	44	37	42	33	39	244	13	9	3	21	18	18
PFr	U	*B. garinii*	human	Germany	1995	48	34	34	44	37	42	33	39	244	13	9	3	21	18	18
20047	153	*B. garinii*	tick ricinus	France	U	40	25	26	36	27	34	25	31	82	15	11	3	22	19	19
CIP 103362	U	*B. garinii*	tick ricinus	France	1992	40	25	26	36	27	34	25	31	82	15	11	3	22	19	19
FNG1-9	U	*B. garinii*	tick uriae	Norway	2017	185	147	134	160	34	170	144	161	575	14	10	3	23	21	21
17-59N1	U	*B. garinii*	tick uriae	Norway	2017	185	147	134	160	34	170	144	161	575	14	10	3	23	21	21
17-58N4	U	*B. garinii*	tick uriae	Norway	2017	185	147	134	160	34	170	144	225	804	14	10	3	23	21	21
17-29-22-5	U	*B. garinii*	tick uriae	Norway	2017	185	147	134	160	34	170	144	161	575	14	10	3	23	21	21
Far04	U	*B. garinii*	puffin	Denmark	U	185	147	134	160	34	170	144	161	575	14	10	3	23	21	21
Ekb704-11	U	*B. garinii*	tick persulcatus	Russia	2011	40	25	26	36	122	34	25	49	364	15	11	3	25	19	19
Ekb701-11	U	*B. garinii*	tick persulcatus	Russia	2011	171	134	78	149	149	120	131	141	431	17	13	5	26	22	22
Ekb712-11	U	*B. garinii*	tick	Russia	2011	67	51	51	61	56	55	47	51	154	36	32	12	27	23	23
HT59	1081	*B. garinii*	tick persulcatus	Japan	U	65	50	49	59	54	54	46	106	367	37	33	11	28	24	24
J-21	U	*B. garinii*	human	Japan	U	58	43	42	52	48	47	39	43	127	38	34	12	29	25	25
PBes	U	*B. garinii*	human	Germany	1989	95	74	34	96	89	78	77	85	251	39	35	7	30	28	28
PKi	U	*B. garinii*	human	Germany	1992	99	77	81	91	88	84	82	33	245	40	36	8	31	30	30
PMe	U	*B. garinii*	human	Germany	1988	112	80	78	99	81	39	79	87	246	18	14	5	32	31	31
PUI	U	*B. garinii*	human	Germany	1999	112	80	78	96	81	39	145	33	578	23	19	6	32	31	31
PMit	U	*B. garinii*	human	Germany	1997	42	27	29	92	29	36	27	33	177	22	18	6	33	31	31
POhm	U	*B. garinii*	human	Germany	1991	42	27	29	38	29	36	27	33	86	22	18	6	34	32	32

Isolate	PubMLST ID	Species	Source	Country	Year of Collection	*clpA*	*clpX*	*nifS*	*pepX*	*pyrG*	*recG*	*rplB*	*uvrA*	ST (MLST)	*ospA* Allele	OspA Variant	OspA IST	OspC Variant	*dbpA*	DbpA Variant
PHc	U	*B. garinii*	human	Germany	1996	42	27	29	38	29	36	27	33	86	22	18	6	35	29	29
PHez	U	*B. garinii*	human	Germany	1994	42	27	29	38	29	36	27	33	86	22	18	6	35	29	29
PSoR	U	*B. garinii*	human	Germany	1989	42	27	29	38	29	36	27	33	86	22	18	6	35	29	29
Malouvrh	U	*B. garinii*	human	Slovenia	2006	43	28	30	90	87	36	28	34	180	25	21	6	36	27	27
PStg	U	*B. garinii*	human	Germany	1996	43	28	30	90	82	36	28	34	179	24	20	6	36	33	27
PNov	U	*B. garinii*	human	Slovenia	1990	43	28	30	90	87	36	28	34	180	25	21	6	36	27	27
Tmsk1187-2013	U	*B. garinii*	tick pavlovskyi	Russia	2013	193	76	34	165	172	180	149	164	614	41	9	3	38	34	34
Tmsk1188-2013	U	*B. garinii*	tick pavlovskyi	Russia	2013	U	157	141	163	173	54	150	165	U	42	37	11	39	35	35
Tmsk1189-2013	U	*B. garinii*	tick	Russia	2013	195	158	142	149	108	181	151	166	616	19	15	5	40	36	36
Tmsk1192-2013	U	*B. garinii*	tick	Russia	2013	112	80	140	96	149	178	147	87	612	20	16	5	41	38	31
Tmsk1193-2013	U	*B. garinii*	tick pavlovskyi	Russia	2013	U	159	U	166	U	182	42	U	U	43	38	3	42	39	39
Tmsk1218-2013	U	*B. garinii*	tick	Russia	2013	196	160	144	91	149	183	152	168	618	21	17	5	43	40	40
17-63	U	*B. garinii*	tick uriae	Norway	2017	U	190	195	36	208	216	181	266	U	44	39	3	44	41	41
935	U	*B. garinii*	tick persulcatus	South Korea	1993	242	203	29	215	221	232	196	216	737	56	49	11	45	42	42
Bb16-16-1	U	*B. burgdorferi*	tick	Canada	2016	7	6	12	1	2	5	5	5	46	94	75	1	48	5	5
Bb16-174	U	*B. burgdorferi*	tick	Canada	2016	7	6	12	1	1	5	5	5	37	7	4	1	48	5	5
Bb16-55-1	U	*B. burgdorferi*	tick	Canada	2016	7	6	12	1	2	5	5	5	46	94	75	1	48	5	5
Bb16-23-2	U	*B. burgdorferi*	tick	Canada	2016	7	6	12	1	2	5	5	5	46	94	75	1	48	U	5
Bb16-15-2	U	*B. burgdorferi*	tick	Canada	2016	7	6	12	1	2	5	5	5	46	94	75	1	48	5	5
Bb16-33-3	U	*B. burgdorferi*	tick	Canada	2016	5	5	4	114	1	15	1	6	302	95	76	1	50	2	2
PBi	155	*B. bavariensis*	human	Germany	U	41	26	27	37	28	35	26	32	84	16	12	4	53	43	43
DK6	U	*B. bavariensis*	human	Denmark	1990	41	26	27	37	28	35	26	32	84	16	12	4	53	43	43
Lubl25	U	*B. bavariensis*	human	Slovenia	1995	41	26	28	37	28	35	26	32	85	16	12	4	53	43	43
PNeb	U	*B. bavariensis*	human	Germany	1988	41	26	27	37	28	35	26	32	84	16	12	4	53	43	43

Isolate	PubMLST ID	Species	Source	Country	Year of Collection	*clpA*	*clpX*	*nifS*	*pepX*	*pyrG*	*recG*	*rplB*	*uvrA*	ST (MLST)	*ospA* Allele	OspA Variant	OspA IST	OspC Variant	*dbpA*	DbpA Variant
PNi	U	*B. bavariensis*	human	Germany	2000	41	26	27	37	28	35	26	32	84	16	12	4	53	43	43
PRab	157	*B. bavariensis*	human	Austria	1994	41	26	27	37	28	35	26	32	84	16	12	4	53	43	43
PRof	U	*B. bavariensis*	human	Germany	1989	41	26	27	37	28	35	26	32	84	16	12	4	53	43	43
PTrob	158	*B. bavariensis*	human	Slovenia	1988	41	26	28	37	28	35	26	32	85	16	12	4	53	43	43
PWin	U	*B. bavariensis*	human	Germany	1987	41	26	27	37	28	35	26	32	84	16	12	4	53	43	43
PZwi	U	*B. bavariensis*	human	Germany	1994	41	26	28	37	28	35	26	32	85	16	12	4	53	43	43
A91S	U	*B. bavariensis*	human	Netherlands	1996	41	26	27	37	28	35	26	32	84	16	12	4	53	43	43
61VB2	U	*B. bavariensis*	tick	Germany	U	41	26	27	37	28	35	26	32	84	16	12	4	53	43	43
PHerl	U	*B. bavariensis*	human	Germany	1989	41	26	27	37	28	35	26	32	84	16	12	4	53	43	43
PLad	U	*B. bavariensis*	human	Germany	2000	41	26	27	37	28	35	26	32	84	16	12	4	53	43	43
PBar	U	*B. bavariensis*	human	Germany	1988	41	26	27	37	28	35	26	32	84	16	12	4	53	43	43
PBN	U	*B. bavariensis*	human	Germany	1999	41	26	27	37	28	35	26	32	84	16	12	4	53	43	43
PBaeII	U	*B. bavariensis*	human	Germany	1990	41	26	U	37	28	35	26	32	U	16	12	4	53	43	43
IBS59	U	*B. bavariensis*	U	U	U	41	26	27	37	28	35	26	32	84	16	12	4	53	43	43
A104S	U	*B. bavariensis*	human	Netherlands	1996	41	26	27	37	28	35	26	32	84	16	12	4	53	43	43
A14S_pubMLST	1001	*B. spielmanii*	human	Netherlands	1992	94	72	71	84	80	75	72	76	159	58	51	13	71	86	86
FP1	U	*B. afzelii*	human	China	U	35	24	23	49	44	27	23	28	106	98	7	2	77	U	U
R9	U	*B. afzelii*	human	China	U	35	24	23	49	44	27	23	28	106	P	P	U	77	U	U
SV1	U	*B. finlandensis*	tick ricinus	Finland	2008	161	8	10	10	9	137	7	129	414	109	87	21	79	U	U
Bb16-57-1	U	*B. burgdorferi*	tick	Canada	2016	18	12	1	11	2	15	1	2	29	92	4	1	80	91	90
Bb16-60-1	U	*B. burgdorferi*	tick	Canada	2016	18	12	1	11	2	15	1	2	29	92	4	1	80	91	90
Bb16-74-1	U	*B. burgdorferi*	tick	Canada	2016	14	1	5	2	2	1	1	10	530	93	48	1	81	U	U
Bb16-135	U	*B. burgdorferi*	tick	Canada	2016	14	1	5	2	2	1	1	10	530	93	48	1	81	U	U

Isolate	PubMLST ID	Species	Source	Country	Year of Collection	*clpA*	*clpX*	*nifS*	*pepX*	*pyrG*	*recG*	*rplB*	*uvrA*	ST (MLST)	*ospA* Allele	OspA Variant	OspA IST	OspC Variant	*dbpA*	DbpA Variant
Bb16-47	U	*B. burgdorferi*	tick	Canada	2016	19	1	5	1	2	1	1	10	30	95	76	1	83	U	U
Bb16-132	U	*B. burgdorferi*	tick	Canada	2016	3	3	2	2	3	4	4	4	221	2	2	1	84	9	9
Bb16-178-2	U	*B. burgdorferi*	tick	Canada	2016	3	3	2	4	3	4	4	4	12	2	2	1	84	9	9
Bb16-17-1	U	*B. burgdorferi*	tick	Canada	2016	3	3	2	2	3	4	4	4	221	2	2	1	84	9	9
Bb16-139	U	*B. burgdorferi*	tick	Canada	2016	3	3	2	2	3	4	4	4	221	2	2	1	84	9	9
Bb16-250	U	*B. burgdorferi*	tick	Canada	2016	3	3	2	4	3	4	4	4	12	2	2	1	84	9	9
Bb16-111	U	*B. burgdorferi*	tick	Canada	2016	18	12	1	11	2	15	1	2	29	7	4	1	85	91	90
Bb16-62	U	*B. burgdorferi*	tick	Canada	2016	18	12	1	11	2	15	1	2	29	7	4	1	85	91	90
Bb16-183	U	*B. burgdorferi*	tick	Canada	2016	8	1	1	1	4	6	1	7	4	2	2	1	86	3	3
Bb16-188	U	*B. burgdorferi*	tick	Canada	2016	8	1	1	1	4	6	1	7	4	2	2	1	86	3	3
Bb16-133	U	*B. burgdorferi*	tick	Canada	2016	4	4	3	3	3	21	6	3	43	5	3	1	87	U	U
Bb16-128	U	*B. burgdorferi*	tick	Canada	2016	4	4	3	3	3	21	6	3	43	5	3	1	87	U	U
Bb16-134	U	*B. burgdorferi*	tick	Canada	2016	4	4	3	3	3	21	6	3	43	5	3	1	87	U	U
Bb16-130	U	*B. burgdorferi*	tick	Canada	2016	4	4	3	3	3	21	6	3	43	5	3	1	87	U	U
Bb16-138	U	*B. burgdorferi*	tick	Canada	2016	4	4	3	3	3	21	6	3	43	5	3	1	87	U	U
Bb16-126	U	*B. burgdorferi*	tick	Canada	2016	4	4	3	3	3	21	6	3	43	2	2	1	87	U	U
Bb16-85	U	*B. burgdorferi*	tick	Canada	2016	20	4	3	3	3	3	3	3	31	5	3	1	88	U	U
Bb16-52	U	*B. burgdorferi*	tick	Canada	2016	20	4	3	3	3	3	3	3	31	5	3	1	88	U	U
Bb16-90-2	U	*B. burgdorferi*	tick	Canada	2016	20	4	3	3	3	18	3	3	229	55	2	1	88	U	U
Bb16-93	U	*B. burgdorferi*	tick	Canada	2016	155	5	4	1	5	197	1	12	641	78	3	1	89	U	U
Bb16-71-2	U	*B. burgdorferi*	tick	Canada	2016	4	4	3	19	3	3	3	3	44	5	3	1	90	U	U
Arh913-2012	U	*B. bavariensis*	tick	Russia	2012	60	45	44	54	50	173	41	45	594	27	23	10	91	44	44
Konnai-17	U	*B. bavariensis*	tick	Japan	2011	60	45	44	54	50	49	41	45	131	32	28	10	91	49	49
PD91	U	*B. bavariensis*	human	China	U	60	45	44	54	50	46	U	45	U	97	77	10	91	44	44
Arh923-2012	U	*B. bavariensis*	tick	Russia	2012	56	42	41	51	47	46	38	42	128	28	24	10	92	45	45

Isolate	PubMLST ID	Species	Source	Country	Year of Collection	*clpA*	*clpX*	*nifS*	*pepX*	*pyrG*	*recG*	*rplB*	*uvrA*	ST (MLST)	*ospA* Allele	OspA Variant	OspA IST	OspC Variant	*dbpA*	DbpA Variant
Prm7564	U	*B. bavariensis*	tick	Russia	U	56	42	41	51	47	149	38	42	433	28	24	10	92	45	45
J-14	1117	*B. bavariensis*	human	Japan	U	60	45	64	54	50	46	108	45	371	29	25	10	93	46	46
FujiP2	1090	*B. bavariensis*	tick	Japan	U	60	45	64	54	50	46	108	45	371	29	25	10	93	46	46
NT24	1087	*B. bavariensis*	tick	Japan	U	60	45	64	54	50	46	108	45	371	29	25	10	93	46	46
J-15	1118	*B. bavariensis*	human	Japan	U	146	105	105	131	131	129	111	115	381	30	26	9	94	47	47
J-20T	U	*B. bavariensis*	human	Japan	U	148	106	107	77	133	130	113	117	383	31	27	10	95	48	48
N346	1082	*B. bavariensis*	tick	Japan	U	153	108	110	135	76	72	115	71	386	33	29	9	96	50	50
Prm965-2013	U	*B. bavariensis*	tick	Russia	2013	144	103	43	130	128	127	110	42	374	34	30	9	97	51	51
Prm7569	U	*B. bavariensis*	tick	Russia	U	144	103	43	130	128	127	110	42	374	34	30	9	97	51	51
Prm7019-12	U	*B. bavariensis*	tick	Russia	2012	317	282	43	282	291	313	270	281	1070	35	31	9	98	52	52
QX-S13	U	*B. yangtzensis*	mouse	China	U	82	61	60	73	68	68	61	64	149	99	78	17	99	U	U
VS116	176	*B. valaisiana*	tick	Switzerland	U	49	35	35	45	38	43	34	40	95	60	53	15	101	U	103
MN14-1539	U	*B. mayonii*	human	USA	2014	218	182	166	191	202	209	174	193	675	57	50	14	102	98	98
MN14-1420_pubMLST	U	*B. mayonii*	human	USA	2014	218	182	166	191	201	209	174	193	674	57	50	14	102	98	98
CO275	U	*B. bissettiae*	U	USA	1994	U	70	U	103	U	U	84	92	U	NH	NH	U	103	U	U
DN127-Cl9-2_P7	U	*B. bissettiae*	tick	USA	U	117	69	68	102	99	74	83	89	U	52	47	19	104	U	U
HO14	U	*B. japonica*	U	Japan	U	178	124	117	142	140	140	121	132	453	48	43	22	105	U	U
Ekb706-11	U	*B. garinii*	tick persulcatus	Russia	2011	171	134	78	149	149	120	131	141	431	17	13	5	P	22	22
Konnai20_cl1	U	*B. garinii*	tick persulcatus	Japan	2011	149	118	108	133	134	131	106	118	384	P	P	U	P	26	26
Tmsk1190-2013	U	*B. garinii*	tick pavlovskyi	Russia	2013	192	80	78	164	171	179	148	33	613	21	17	5	P	37	37

Abbreviations: ST, sequence type; MLST, multilocus sequence type; IST, *in silico* type; P, partial; U, unknown; NH, no hit

Table S3. Pairwise amino acid sequence identities of seven immune-evasion genes in Dutch *Borrelia* isolates belonging to OspA IST1, IST2, and IST4-6.

Locus	Size (bp)	Description	IST1 SEQID to B31[a]	IST2 SEQID to B31[a]	IST4 SEQID to B31[a]	IST5 SEQID to B31[a]	IST6 SEQID to B31[a]
BB0238	789	hypothetical protein	98.9-100	89.8-90.2	89	90.1	89.7-90.1
BB0323	1134	*B. burgdorferi* ss predicted coding region	97.1-99.5	90-90.5	88.7	87.9	87.9-88.2
BB0365	585	lipoprotein LA7	98.5-100	99.0-99.5	71.4	73.5	73.0-73.5
BB0405	612	hypothetical protein	99.5-100	86.8-88.7	94.6	93.1	93.6-94.1
BB0406	612	hypothetical protein	98.5-100	91.2-91.7	90.2	98.7	89.2-89.7
BB0744	2103	antigen, p83/100	98-100	Truncated	88.3	88	87.0-87.5
BB_K32	1065	hypothetical protein	97.8-99.4	71.0-71.3	82.3	90	86.9-89.2

[a] as compared to the *B. burgdorferi* ss B31 reference genome

Abbreviations: bp, base pairs; IST, *in silico* type

References

1. Coipan EC, Jahfari S, Fonville M, Oei GA, Spanjaard L, Takumi K, Hovius JW, Sprong H: **Imbalanced presence of Borrelia burgdorferi s.l. multilocus sequence types in clinical manifestations of Lyme borreliosis**. *Infection, genetics and evolution : journal of molecular epidemiology and evolutionary genetics in infectious diseases* 2016, **42**:66-76.
